# Ecological remediation strategy for urban brownfield renewal in Sichuan Province, China: a health risk evaluation perspective

**DOI:** 10.1038/s41598-022-08268-z

**Published:** 2022-03-11

**Authors:** Weike Zhao, Yuanpei Liao, Shengqiu Zhou, Bo Zhou

**Affiliations:** College of Architecture and Environment, Sichuan University, Chengdu, 610065 China

**Keywords:** Environmental impact, Environmental sciences, Solid Earth sciences

## Abstract

Urban brownfield sites are abandoned industrial land and their redevelopment may be affected by environmental pollution, as the latter may pose health risks for residents. In this study, six heavy metals (Pb, As, Cr, Zn, Ni, and Cu) were examined from 87 soil samples extracted from four land use types (industrial area, residential/commercial area, traffic area, and agricultural area) in the Mianyang thermal power plant area, Sichuan Province, China. The soil contamination and environmental risk were evaluated using the single factor index, geo-accumulation index and Human Health Risk Evaluation. ArcGIS was used to map out the spatial distribution of heavy metal concentrations and environmental risk. The results of these analyses have indicated that different land use types have significant effects on the heavy metal contamination of soil. There are 10 non-carcinogenic risk areas of heavy metals in industrial land, while in the other three types there are 9 non-carcinogenic risk areas of heavy metals. Under the brownfield renewal planning, the present study scheme provides an effective method of discernment for ecological remediation of soil heavy metals. In addition, it can aid brownfield in finding different remediation strategies with economic benefits for different risk levels of human health.

## Introduction

Along with urban development and industrial innovation, many countries today are facing two important challenges related to urban construction. The first issue of relevance is the remediation of large-scale environmental contamination caused by industrial activities, while the second concerns the renewal of urban brownfield sites. Urban areas have a significant number of abandoned, or unused industrial and commercial land with facilities, and these industrial and commercial lands are classified as brownfield^1^. Urban expansion may have been affected by environmental contamination. As a result, it may have reduced the value of land and hampered local development^2^. Analysis of brownfield site remediation was initiated in Western Europe and the United States in the early 1970s^3^. Currently, 340,000 brownfield sites in the European Union are expected to be contaminated and will require environmental restoration before redevelopment^4^. Moreover, there are more than 450,000 brownfield sites in the United States which could be redeveloped^5^. China has 120 old industrial cities^6^, and over 2,615,400 hectares of abandoned industrial land^7^. The significant number of brownfield sites suitable for redevelopment may be able to alleviate urban land shortages and environmental pressure, while potentially stimulating economic growth after treatment as well^8^. However, due to industrial production, human activities and transportation, soil contamination in brownfield sites is complex and dynamic, and will change along with land use^9^. Therefore, the renewal of brownfield sites is more challenging than the development of ordinary land. In order to determine an ecological remediation strategy and to improve land value, a detailed study of environmental contamination with respect to urban brownfield sites is required.

As the final location of most pollutants, polluted soils can endanger human health. The increased content of heavy metals in brownfield sites does not only directly affect the physical and chemical properties of the soil, but threatens human health through the food chain, as well as respiratory and skin contact^10^. People can be exposed to contaminated soil through oral ingestion of soil, skin contact, and inhalation of airborne particles from soil^26^. Heavy metals elements can be divided into essential and non-essential metals according to their presence in the human body and their significance for it. Essential metal elements are a group that must be present in the human body, such as Cu, Zn and Cr^11^. Their absence may cause serious damage to bodily functions. The recommended dose of Zn is around 15 mg/day^12^. If consumed in excess, it may cause nausea, vomiting, and stomach cramps^13^. Non-essential metal elements are those which are optionally present in the human body as well as in the food, water, and air^11^. An example of a non-essential metal is Pb. Pb contamination has been recorded to damage nerves, bones, and the immune system^13^. Therefore, health risk evaluation of heavy metal contaminated soil is a key aspect of brownfield ecological remediation and has attracted the attention of scholars around the world^14^. The potential ecological risk index, hazard index, and carcinogenic risk can be used to evaluate the environmental risk of different types of land use and can provide data support for different land types of management policies^15^. The geo-accumulation index, the tucker 3 model, and the health risk evaluation aided in the analysis of heavy metal pollution and possible health risks^16^. The principal component analysis (PCA) and cluster analysis (CA) were employed to examine the sources and levels of heavy metals contamination in soil^17^. In addition, spatial interpolation was able to reflect the spatial distribution of harmful substances in soil, as well as estimate the variables of non-sampled locations^18^. Based on the geographic detection methods and Human Health Risk Evaluation (HHRE), it is possible to estimate and map the continuous surfaces of carcinogenic and non-carcinogenic risks caused by heavy metals^19^. The Grey Relational Analysis (GRA) model with Geographic Information System (GIS) was used by scholars to produce geochemical mapping and to analyze heavy metal pollution levels in soi^20^. In China, rapid urbanization has led to an abundance of demands for land resources and increased concern for public health security. We refer to the PRC method, suggested by the Ministry of Ecology and Environment in order to evaluate soil health risks. Our research represents important data for ecological remediation of brownfield sites and can support the creation of the sustainable urban environment.

Due to the geographical differences of soil pollution in brownfield sites, traditional data evaluation is difficult for achieving reasonable ecological remediation. Many scholars have used statistics, such as PCA and CA, in order to evaluate the heavy metal pollution of soil. However, in the context of landscape planning, the environmental evaluation of the soil before brownfield renewal needs to analyze the geographical differences of heavy metal risk by using statistics combined with GIS. This is important to obtain ecological remediation strategies with economic benefits. The research objective in the present paper was the Mianyang City thermal power plant area. The case was built early and had great industrial significance. After its abandonment, it is now a government-designated urban renewal project. Moreover, the primary objective was to evaluate the soil pollution and health risks of different land use types through Factor Analysis and ArcGIS. We created the ecological remediation strategies for polluted brownfield sites based on the results of environmental evaluation and the method of landscape planning. The suggested strategies may be utilized to change and improve the biological and physical conditions of degraded brownfield sites, including restoration of the natural land environment, elimination of pollution defects, and reconstruction of the use functions^21^. In addition, the results of this study will generate new ideas for the landscape renewal of urban brownfield sites from the perspective of environmental science and urban planning.

## Materials and methods

### Study area

**Figure 1:**
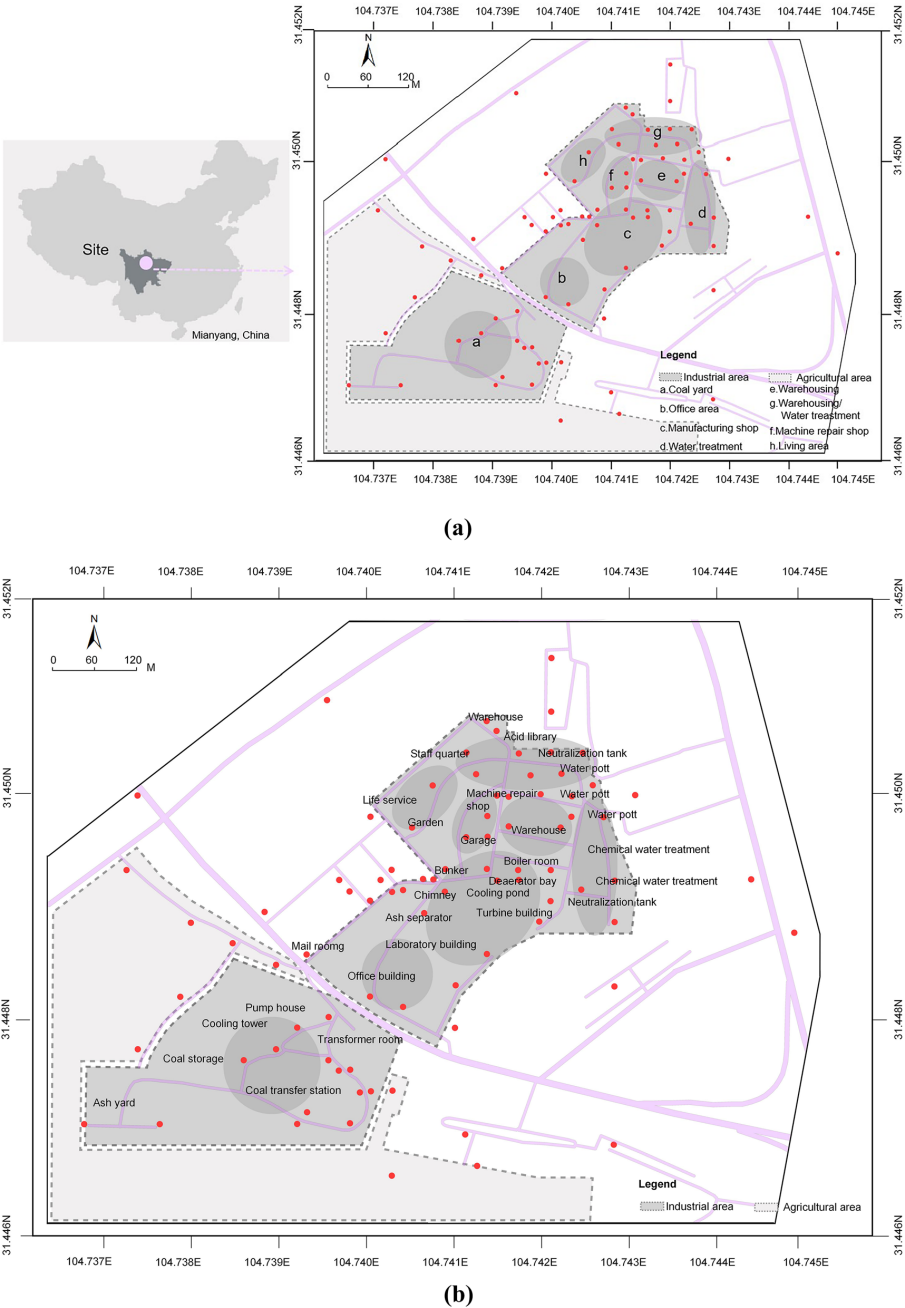
The study area: **(a)** Location of the study area; **(b)** Functional layout of the industrial area. (Note: The base map was designed by the author through referring to Baidu Map, https://map.baidu.com and painting the City boundary and urban road network with Adobe Photoshop CS6.)

The Mianyang thermal power plant, which is located in Mianyang City, Sichuan Province, was selected as the study area (Fig. 1a). It currently faces pressures in relation to soil pollution and urban renewal. The examined region has an area of 569,100 m^2^, of which 20.06% makes up industrial land, 13.61% is agricultural, 5.47% is traffic land. The rest of the region is considered residential/commercial land. Furthermore, the industrial area may be divided into office, production, power, storage, and other types of spaces (Fig. 1b). The Mianyang thermal power plant area is representative of brownfield renewal in China. Mianyang City is the second largest city in Sichuan Province, with a strong industrial foundation. It is currently included in the Chinese government’s transformation plan of old industrial bases^6^. The city’s thermal power plant was established in the 1970s, providing heat and electricity for 70% of urban residents and had an irreplaceable role in urban industrial activities. However, the power plant was decommissioned in 2016 and the resulting brownfield site is presently faced with a number of serious environmental problems. As this area was included in the urban renewal projects of local government in 2020^22^, there is an urgent need for the evaluation of soil pollution, as it is a prerequisite for brownfield renewal.

### Sampling and analysis

In order to analyze heavy metal soil contamination across the area, soil samples were collected from different land use types. The sampling points were arranged as shown in Fig. 1. The soil samples collected were mainly collected those representing different production activities in the industrial area. It should be noted that a few samples from the residential/commercial, traffic and agricultural areas were collected as well. Therefore, the spatial distribution of samples was balanced out. Despite limited accessibility to some sites, a total of 87 soil samples were collected in January 2021. Due to increased heating in winter, contaminants in the thermal power plant and its surrounding areas may have increased with seasonal change. After the sampling points were recorded by the GPS measuring instrument, the stainless steel soil sampler was used to collect the soil. Moreover, a composite topsoil sample was mixed by 5 samples nearby (depth = 0–20 cm). The samples were stored in Poly Vinyl Chloride (PVC) packages with sample information labels. They were brought to the laboratory on the day of sampling.

The soil samples were initially air-dried for 24 h, before being passed through a 2-mm sieve that removed stones and plant debris. Afterward, the samples were mechanically mixed, packed into PVC packages, and labeled with sample information to ensure the uniformity of subsequent analyses. Lead (Pb), chromium (Cr), zinc (Zn), nickel (Ni), and cuprum (Cu) concentrations were determined using flame atomic absorption spectrophotometry (PinAAcle 900H, PerkinElmer). In addition, arsenic (As) concentrations were determined using atomic fluorescence spectrometry (BOEN-35851, Fairborn). Duplicate samples were simultaneously analyzed for approximately 20% of soil samples in the assays, using a standard deviation range of 5%.

The validity and accuracy of the data have been ensured by the self-check of the instrument system and the measurement of standard soil samples. The determination results of the standard material for soil composition analysis, purchased from the Institute of Geophysical and Geochemical Exploration, Chinese Academy of Geological Sciences (IGGE) showed that the recovery of each element was 90–120%.

### Risk evaluation

The six heavy metals Pb, As, Cr, Zn, Ni, and Cu selected in this experiment have the features of persistence, latency, migration, and accumulation in soli. Due to the fact that the heavy metal pollution of soil is harmful to animals, plants, and humans, it is necessary to understand the environmental status through the evaluation of soil contamination using single factor index method, geo-accumulation index method and HHRE. This will provide a basis for the reuse of land resources as well as soil pollution control. In addition, the combination of Factor Analysis and geo-statistics can compensate the shortcomings of statistical methods in the study on soil pollution using spatial differences.

#### Single factor index method

The single factor index ($P_{i}$) is a commonly used method for calculating the health quality of soil. This index was calculated as:

1 $$p_{i} = \frac{{C_{i} }}{{S_{i} }}$$

In the above formula, C_*i*_ represents the measured concentration of element i (mg kg^−1^), while *S*_*i*_ is the standard value of element i in the soil (mg kg^−1^) according to the Chinese standard^23^. The evaluation standard of the single factor index method is shown in Supplementary Table S1. Furthermore, $P_{i}$ ≤ 1 represents soil without pollution, while $P_{i}$ > 5 represents severely polluted soil. The larger the value of $P_{i}$, the more serious the soil pollution.

#### Geo-accumulation index method

The geo-accumulation index ($I_{geo}$) is used to quantitatively examine the degree of heavy metal pollution in sediments and soil^24^. This index was calculated as:

2 $$I_{geo} = \log_{2} \left( {\frac{{C_{n} }}{{1.5B_{n} }}} \right)$$

In the above formula, $C_{n}$ represents the measured concentration of element n (mg kg^−1^), while $B_{n}$ is the geochemical background value of heavy metals in the soil (mg kg^−1^) according to the standard value of Sichuan Province, China^25^. Furthermore, $I_{geo}$ < 0 indicates soil without pollution, while 5 < $I_{geo}$ ≤ 6 represents severely polluted soil. Therefore, the larger the value of $I_{geo}$, the greater level of soil pollution.

#### Human health risk evaluation (HHRE)

The HHRE was formulated to explore the potential risk levels of human exposure to soil pollutants. In this study, the focus has been placed on evaluating the Hazard Quotient (HQ) and Carcinogenic Risk (CR) of heavy metals in the soil. HQ is used to characterize the level of harm caused by non-carcinogenic pollutants through a single or multiple pathways^26^. CR is used to examine the probability of carcinogenic diseases or injury caused by carcinogenic pollutants^26^. Firstly, the corresponding land risk coefficient was identified according to land use types. Secondly, by considering the actual situation of the study area, HQ and CR of oral ingestion ($HQ_{ois}$), dermal contact ($HQ_{dcs}$), and particulate inhalation ($HQ_{pis}$) were calculated. Finally, in accordance with the acceptability level of CR of a single heavy metal in soil being ≤ 10^–6^, and the acceptability level HQ being ≤ 1, the possible health risk of the site was evaluated^27^. According to the Technical Guidelines for Risk Assessment of Contaminated Sites^25^, the three exposure pathways were calculated as:

3 $$HQ_{ois} = \frac{{OSIR \times ED \times EF \times C_{sur} \times ABS_{o} \times 10^{ - 6} }}{{RfD_{o} \times BW \times AT_{nc} \times SAF}}$$

In formula (3), $HQ_{ois}$ represents the hazard quotient from oral ingestion of soil, while $OSIR$ is the daily oral ingestion rate of soils (children (100 mg d^−1^), adults (200 mg d^−1^))_;_ $ED$ is the exposure duration (children (6a)_,_ adults (24a)), while $EF$ is the exposure frequency, children (350 ${\text{d}}\;{\text{a}}^{ - 1}$), adults (350 ${\text{d}}\;{\text{a}}^{ - 1}$ ). Moreover, the symbol $C_{{{\text{su}}r}}$ stands for the measured concentration of contaminants in the surface soil (mg kg^−1^) derived from the site investigation and $ABS_{O}$ is the absorption factor of oral ingestion (1). The symbol $BW$ is the average body weight (children (15.9 kg), adults (56.8 kg)), while $RfD_{o}$ represents the reference dose for oral ingestion (mg kg^−1^ d^−1^), $RfD_{o}$ of As is 3.00E^-04^ mg kg^−1^ d^−1^, $RfD_{o}$ of Cr is 3.00E^-03^ mg kg^−1^ d^−1^, $RfD_{o}$ of Zn is 3.00E^-01^ mg kg^−1^ d^−1^, $RfD_{o}$ of Pb is 3.50E^-03^ mg kg^−1^ d^−1^, $RfD_{o}$ of Cu is 4.00E^-02^ mg kg^−1^ d^−1^, $RfD_{o}$ of Ni is 2.00E^-02^ mg kg^−1^ d^−1^. Lastly, $AT{}_{nc}$ is the average time for non-carcinogenic effect, (children (2190 d), adults (8760 d)) and *SAF* is the distribution coefficient of the reference dose (0.20).

4 $$HQ_{dcs} = \frac{{239 \times H^{0.417} \times BW^{0.517} \times SER \times SSAR \times EF \times E_{v} \times C_{sur} \times ABS_{d} \times 10^{ - 6} }}{{RfD_{o} \times BW \times AT_{nc} \times ABS_{gi} \times SAF}}$$

Within the above formula, $HQ_{dcs}$ is the hazard quotient of dermal contact with a soil and $H$ is the average height (children (99.4 cm), adults (156.3 cm)). Additionally, the symbol ${\text{SER}}$ represents the skin exposure ratio (children (0.36), adults (0.32)), while ${\text{SSAR}}$ stands for the adherence rate of soil on skin (children (0.2 mg cm^−2^), adults (0.07 mg cm^−2^)). $E_{v}$ is the daily exposure frequency of dermal contact (1 time d^−1^ ) and $ABS_{d}$ is the absorption factor of dermal contact (chemically specific), $ABS_{d}$ of As is 3.00E^-02^, $ABS_{d}$ of Pb is 3.52E^-03^, $ABS_{d}$ of Zn is 1.00E^-03^, $ABS_{d}$ of Cr is 1.00E^-03^, $ABS_{d}$ of Ni is 2.00E^-02^. Finally, $ABS_{gi}$ represents the absorption factor of the digestive tract (chemically specific), $ABS_{gi}$ of As is 1, $ABS_{gi}$ of Cr is 0.013, $ABS_{gi}$ of Ni is 0.04.

5 $$HQ_{pis} = \frac{{PM_{10} \times DAIR_{c} \times ED \times PIAF \times \left( {fspo \times EFO + fspi \times EFI} \right) \times C_{sur} \times BW_{a} \times 10^{ - 6} }}{{RfC \times DAIR_{a} \times AT_{nc} \times SAF \times BW_{c} }}$$

Within Formula (5), $HQ_{pis}$ is the hazard quotient of inhaled soil particulates and $PM_{10}$ is the content of inhalable particulates in ambient air (0.15 mg m^−3^). Furthermore, $DAIR$ is the daily air inhalation rate (children (7.5 m^3^ d^−1^)_,_ adults (14.5 m^3^ d^−1^)), while $PLAF$ represents the retention fraction of inhaled particulates in body (0.75). The ${\text{fspo}}$ represents the fraction of soil-bome particulates in outdoor air (0.5), while $fspi$ is the fraction of soil-bome particulates in indoor air (0.8). Lastly, $EFO$ symbolizes the outdoor exposure frequency (children (87.5 d a^−1^)_,_ adults (87.5 d a^−1^)), while $EFI$ is the indoor exposure frequency (children (262.5 d a^−1^)_,_ adults (262.5 d a^−1^)) and $RfC$ is the reference concentration of respiratory inhalation (mg m^−3^), $RfC$ of As is 1.50E^-05^ mg m^−3^, $RfC$ of Cr is 1.00E^-04^ mg m^−3^, $RfC$ of Ni is 9.00E^-05^ mg m^−3^.

6 $$CR = OISER \times C_{sur} \times SF$$

Finally, in Formula (6), $CR$ stands for the carcinogenic risk from soil ingestion (oral ingestion, dermal contact, or inhalation), while *OISER* is the exposed quantity of soil exposure through oral ingestion, dermal contact, or inhalation (mg kg^−1^ d^−1^). *OISER* (oral ingestion) is 1.568E^-06^, *OISER* (dermal contact) is 4.459E^-06^, and *OISER* (inhalation) is 9.729E^-09^. Lastly, *SF* is the carcinogenic slope factor (kg d mg^−1^), SF of As is 1.50E ^+00^ kg d mg^−1^, SF of Ni is 1.02E ^+00^ kg d mg^−1^ and SF of Cr is 5.00E^-01^ kg d mg^−1^.

### Geospatial distribution

#### The use of geo-statistics

Geo-statistics^28^ provides a theoretical basis for studying the geospatial distribution of heavy metals in soil. Although this method is currently employed in environmental research to determine the characteristics of contaminated sites^29^, combining it with landscape planning and ecological remediation in urban brownfield sites requires further consideration. In the present paper, a soil database was constructed using the GIS (ArcGIS, Version 10.3, ESRI), into which geographical coordinates of soil samples, heavy metals concentrations, and potential health risks were input (Supplementary Table S2, Supplementary Table S5). The possible distribution of heavy metal concentrations and of potential health risks in the study area were determined using the Inverse Distance Weighting method.

#### Factor analysis method

Factor Analysis^30^ is a method used for determining the correlation and principal components of heavy metals in soil. This analysis can calculate the weight of heavy metals in contaminated soil and the weight is used for the superposition of spatial information of human health risk evaluation of various heavy metals. Moreover, factor analysis is the practice of condensing many variables into only a few because it groups together highly related variables into a single category. In comparison to Principal Component Analysis (PCA), Factor Analysis has the benefit of taking the strength of the correlation into account. Thus, it solves the computation obstacle of PCA through the rotation of the factor axis. In this study, the weight of heavy metals was calculated using the following steps:

Step 1: Data standardization was obtained as:

7 $$y_{ij} = \frac{{x_{ij} - \overline{x}_{j} }}{{s_{j} }}$$

where $x_{ij}$ represents the $i$th data of the $j$th factor, where $j$ = 1,2…$n$, $\overline{x}_{j} = \frac{1}{n}\sum\limits_{{{\text{j}} = 1}}^{n} {x_{ij} }$, $s_{j} = \sqrt {\frac{1}{n - 1}\sum\limits_{j = 1}^{n} {\left( {x_{ij} - \overline{x}_{ij} } \right)^{2} } }$.

Step 2: The variance contribution rate of the common factor *F*_m_ was calculated as:

8 $$F_{{\text{m}}} = u{}_{1m}x_{1} + u_{2m} x_{2} + \cdots + u_{jm} x_{j}$$

where *F*_m_ is $m$ represents the common factors obtained according to the principle that the cumulative variance proportion is greater than 60%. Furthermore, $u_{jm}$ symbolizes the coefficient vector in the decision matrix, $u_{jm} = \frac{{f_{jm} }}{{\sqrt {\lambda_{m} } }}$, in which $f_{jm}$ stands for the factor loading and $\lambda_{m}$ is the eigenvalue corresponding to the *m*th common factor.

Step 3: The score coefficient $x_{j}$ of factors was determined as:

9 $$x_{j} = \frac{{\sum {F_{m} \cdot u_{jm} } }}{{\sum {F_{m} } }}$$

Step 4: The factor weight was then normalized to obtain $w_{j}$, as:

10 $$w_{{\text{j}}} = \frac{{x_{j} }}{{\sum {x_{j} } }}$$

### Ethics statement

Our study is based on open source data, so there are no ethical issues and other conflicts of interest.

### Consent to participate

Written informed consent for participation was obtained from all participants.

### Consent to publish

Written informed consent for publication was obtained from all participants.

## Results

### Soil heavy metal concentrations

#### Descriptive analysis of heavy metals

The soil sample data was brought into IBM SPSS Statistics 26.0, where the variability ranges, median values, and standard deviations (SD) of Pb, As, Cr, Zn, Ni, and Cu concentrations were obtained by descriptive statistics (Table 1). The statistical test of outlier was based on the maximum and minimum values. It was used to determine whether the value of a variable exceeded a reasonable range. No outliers were identified in this experimental data. Furthermore, the results indicate that land use types have a significant effect on soil heavy metal concentration. Heavy metal concentrations in the four land use types were recorded to be 3.48 (Pb), 1.26 (As), 3.31 (Cr), 2.45 (Zn), 3.68 (Ni), and 4.13 (Cu) times greater than the background values of Sichuan Province. Through the comparison of heavy metal concentrations in the four land use types, it was observed that As and Ni had the highest mean concentrations, with a value of 12.37 and 87.91, respectively in the industrial area. In addition, the mean concentrations of Pb, Cr, Zn, Ni, and Cu were lowest in the agricultural area, with a value of 42.38, 143, 158.63, 69.38, and 45, respectively. It should also be noted that the maximum concentrations of Pb, As, Zn, Ni, and Cu were recorded in the industrial area, with a value of 392, 21, 674, 303 and 801, respectively. Lastly, it should be noted that the SD results were significantly different, as they indicated that human activities associated with soil samples are highly heterogeneous^31^.

**Table 1:** Soil heavy metal concentrations in the four land use areas (mg kg^-1^).

Land use type		Pb	As	Cr	Zn	Ni	Cu
Industrial area	Mean	74.37	12.37	167.21	209.54	87.91	78.82
	Median	51.00	12.10	161.00	167.00	85.00	45.00
	Max	392.00	21.00	268.00	674.00	303.00	801.00
	Min	17.00	5.39	57.00	115.00	47.00	27.00
	SD	70.95	2.69	41.32	103.51	33.29	114.32
Residential and commercial area	Mean	98.43	9.37	183.50	239.86	77.79	81.79
	Median	49.50	9.52	157.50	205.50	78.50	39.00
	Max	389.00	11.40	400.00	587.00	99.00	608.00
	Min	26.00	6.65	120.00	100.00	62.00	26.00
	SD	110.45	1.36	82.38	147.02	10.67	151.87
Traffic area	Mean	54.13	9.52	161.00	197.00	80.13	77.50
	Median	43.00	9.19	167.00	171.00	83.00	62.00
	Max	150.00	11.10	197.00	354.00	101.00	222.00
	Min	26.00	8.82	98.00	123.00	61.00	33.00
	SD	39.82	0.79	35.47	74.91	15.13	60.16
Agricultural area	Mean	42.38	11.40	143.00	158.63	69.38	45.00
	Median	34.50	9.13	147.00	157.50	73.00	37.50
	Max	73.00	23.20	205.00	211.00	81.00	76.00
	Min	26.00	8.38	92.00	121.00	48.00	33.00
	SD	17.78	5.10	34.57	30.70	10.11	17.25
Background values	Mean	21.40	9.80	50.50	85.50	23.90	19.10

#### Spatial distribution of heavy metals

**Figure 2:**
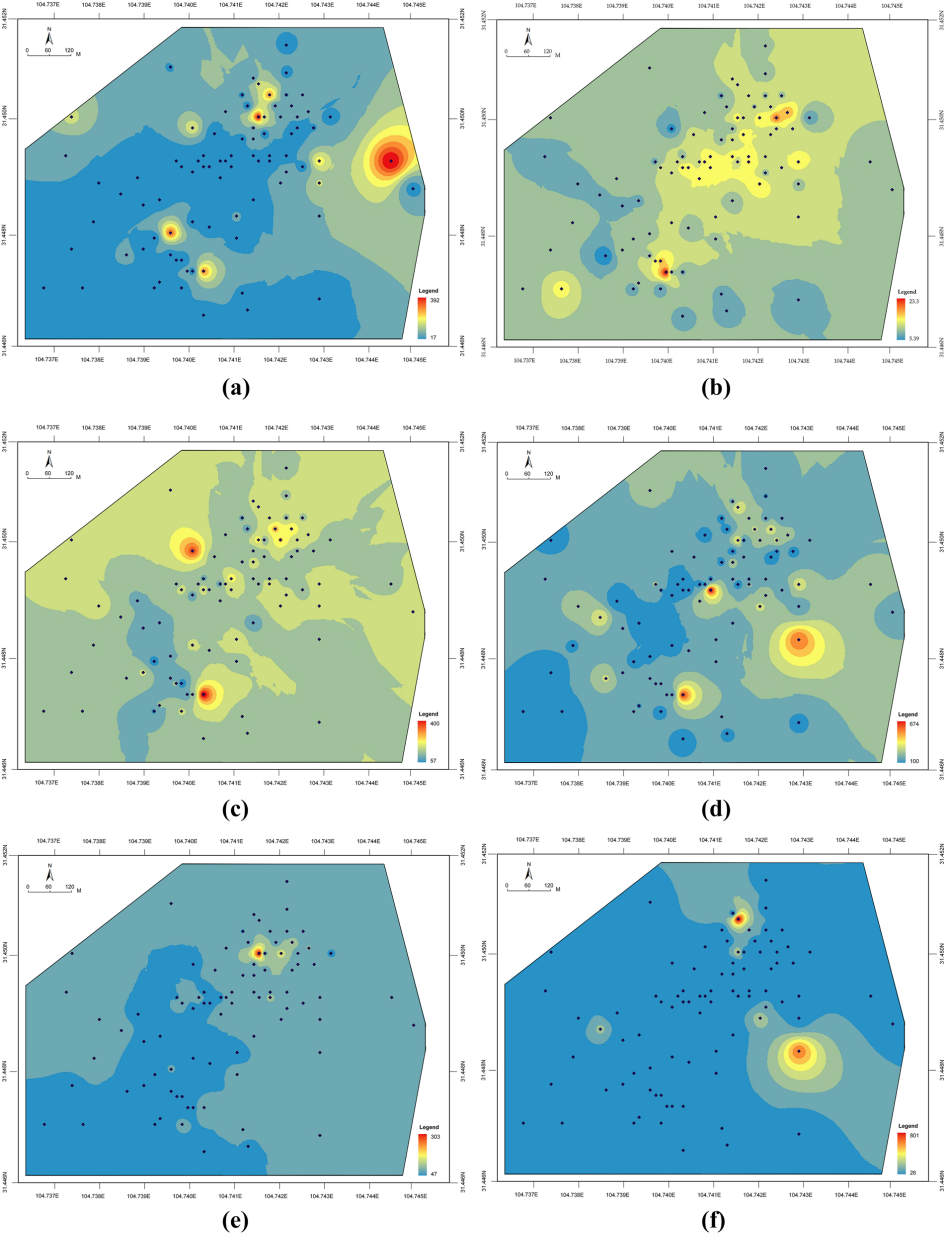
Spatial distribution of heavy metal concentrations in soil: (**a**) Pb; (**b**) As; (**c**) Cr; (**d**) Zn; (**e**) Ni; (**f**) Cu; The color range spanning from blue across yellow to red, represents a spectrum of low to high concentration of heavy metals in the soil. (Note: ArcGIS, Version 10.3, ESRI was used to create the map in this figure.)

The spatial distribution of Pb, As, Cr, Zn, Ni, and Cu concentrations within the study area (Fig. 2) indicates significant differences in the distribution of heavy metal concentration in geographical areas. From a comprehensive distribution perspective, low concentrations were observed in the northwestern region, while high concentrations were recorded in the southeastern one. Nevertheless, concentrations of Pb, As, Cr, and Zn were shown to be high in some places in the northwest, possibly as a result of industrial activities. Furthermore, from the perspective of spatial differences, Pb, As, Cr, and Zn concentrations showed a clear geographic variation, while Ni and Cu recorded a minor geographic variation. Samples containing the maximum concentrations of both Pb and Ni were both located near the machine repair workshop in the industrial area. Moreover, the maximum concentration of As was located at the intersection of the industrial and agricultural areas, where agricultural land adjoins the coal transfer station. The maximum concentration of Cr was located in the residential area, near a coal yard. Heavy metal contamination in this area may be related to contaminated rainwater runoff and air flow. Lastly, the maximum concentration of Zn was located near the main workshop, while the maximum concentration of Cu was found to be in the chemical water treatment area.

#### Correlation of heavy metals

**Figure 3:**
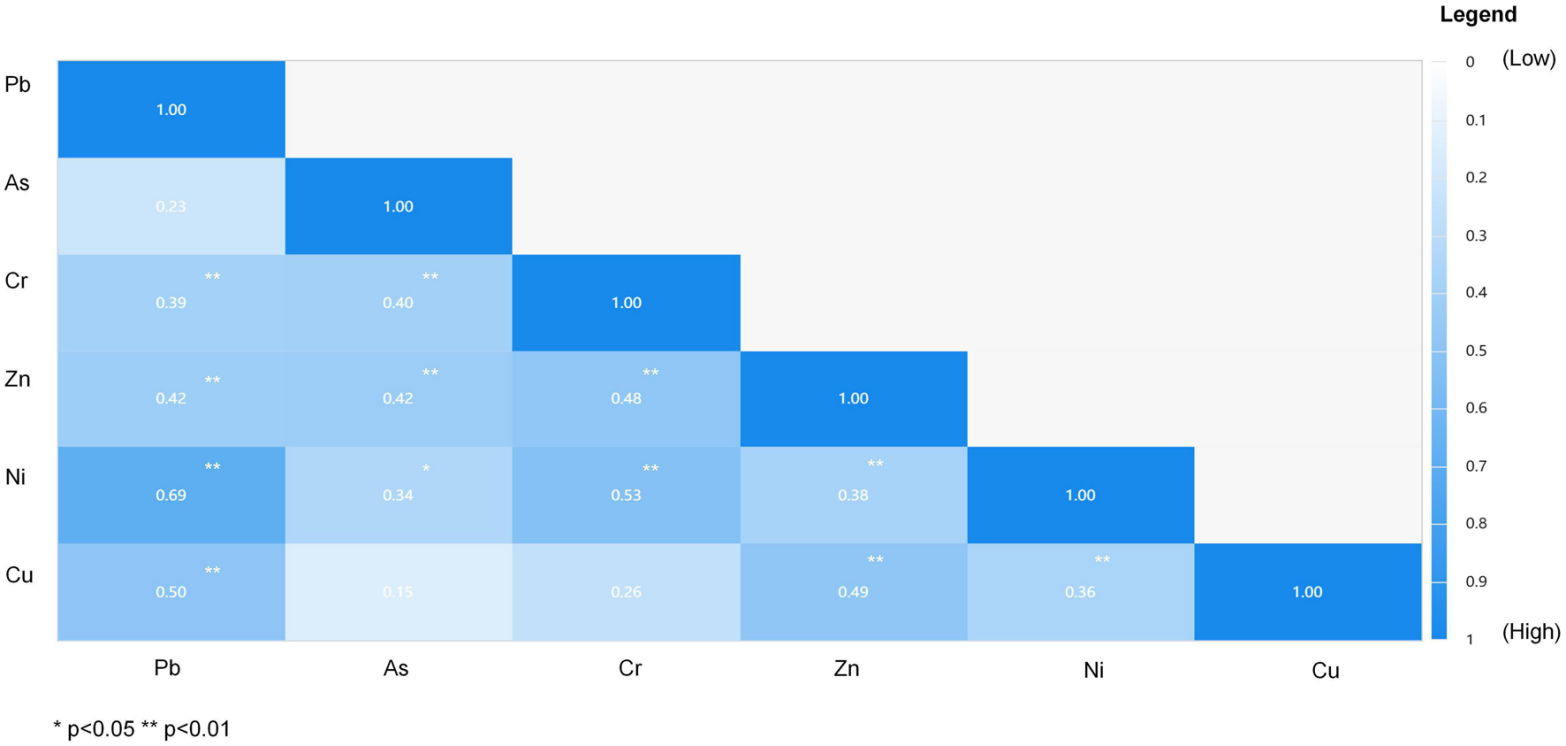
Pearson coefficient correlation of soil heavy metals: blue from light to dark represents the correlation from low to high. (Note: SPSS 26.0 was used to create this figure.)

Correlation and Factor Analysis can effectively indicate the relationship degree between the six heavy metals found in the soil (Fig. 3) and the classification of pollution sources. Descriptive statistics was employed to test the normal distribution of the data. If the absolute value of kurtosis appeared to be less than 10 and the absolute value of skewness is less than 3, the data were accepted as normal distribution. The kurtosis and skewness of the experimental data are illustrated in Supplementary Table S3. The experimental data met the normal distribution. It is appropriate to use Pearson correlation coefficient for the correlation analysis of the six heavy metals. When *P* < 0.01, Pb is correlated with Cr (r = 0.39), Zn (r = 0.42), Ni (r = 0.69), and Cu (r = 0.50). Furthermore, As has a positive correlation with Cr (r = 0.40) and Zn (r = 0.42), while Cr has a significant positive correlation with Zn (r = 0.48) and Ni (r = 0.53). Additionally, Zn is observed to correlate with Ni (r = 0.38) and Cu (r = 0.49). Among the aforementioned, Pb–Ni (0.69) and Cr–Ni (0.53) recorded the highest correlation coefficients, indicating that Pb, Ni, and Cr may be of similar origin.

The interpretation rate of variance, factor loads, and weight results of the factors can be obtained by substituting the concentrations of the six heavy metals into formulas (7)–(10). The Factor Analysis revealed that there were two eigenvalues greater than 1, and their accumulation contribution rate reached 62.41%, as shown in Table 2 and Supplementary Figure S1. Moreover, factor loads for the six heavy metals were obtained through the use of varimax rotation (Table 3). The results indicate that Pb, Cr, Zn, and Ni may have the same pollution source. Additionally, their load coefficients for the main factor 1 are 0.80, 0.80, 0.67, and 0.67, respectively (Supplementary Fig. S2). As the heavy metals As and Cu have load coefficients of 0.86 and 0.57for the main factor 2, they also may have the same pollution source (Supplementary Fig. S2). Furthermore, it should be noted that the Factor Analysis results were consistent with those recorded using Pearson coefficient correlation analysis. Pollution sources may be of two types: (i) coal combustion and steel equipment operation during power generation, and (ii) vehicle exhaust emissions. To comprehensively evaluate health risk, the weight of Pb, As, Cr, Zn, Ni, and Cu were calculated using the "component score coefficient matrix" to be 0.17, 0.12, 0.15, 0.20, 0.19, and 0.17, respectively (Table 4).

**Table 2:** Interpretation rate of variance.

Factor number	1	2	3	4	5	6
Characteristic root	2.69	1.05	0.84	0.65	0.46	0.30
Variance interpretation rate (%)	44.88	17.53	14.07	10.78	7.67	5.07
Accumulation (%)	44.88	62.41	76.48	87.27	94.93	100.00

**Table 3:** Factor loads after rotation.

Factor number	Pb	As	Cr	Zn	Ni	Cu
Main factor 1	0.80	−0.09	0.80	0.67	0.67	0.41
Main factor 2	0.05	0.86	−0.09	0.43	0.37	0.57

**Table 4:** Linear combination coefficients and weight results.

Name	Main factor 1	Main factor 2	Scoring coefficient	Weight
Variance interpretation rate (%)	0.39	0.23		
Pb	0.52	0.05	0.34	0.17
As	−0.06	0.73	0.24	0.12
Cr	0.52	−0.07	0.30	0.15
Zn	0.44	0.36	0.41	0.20
Ni	0.44	0.31	0.39	0.19
Cu	0.27	0.49	0.35	0.17

Based on the analysis of heavy metal concentrations in the soil of the Mianyang thermal power plant area, it was concluded that the evaluation of soil contamination and the health risks it poses is necessary for ecological remediation of soil in this brownfield site.

### HHRE of heavy metals in contaminated soil

#### Contamination evaluation of soil

**Figure 4:**
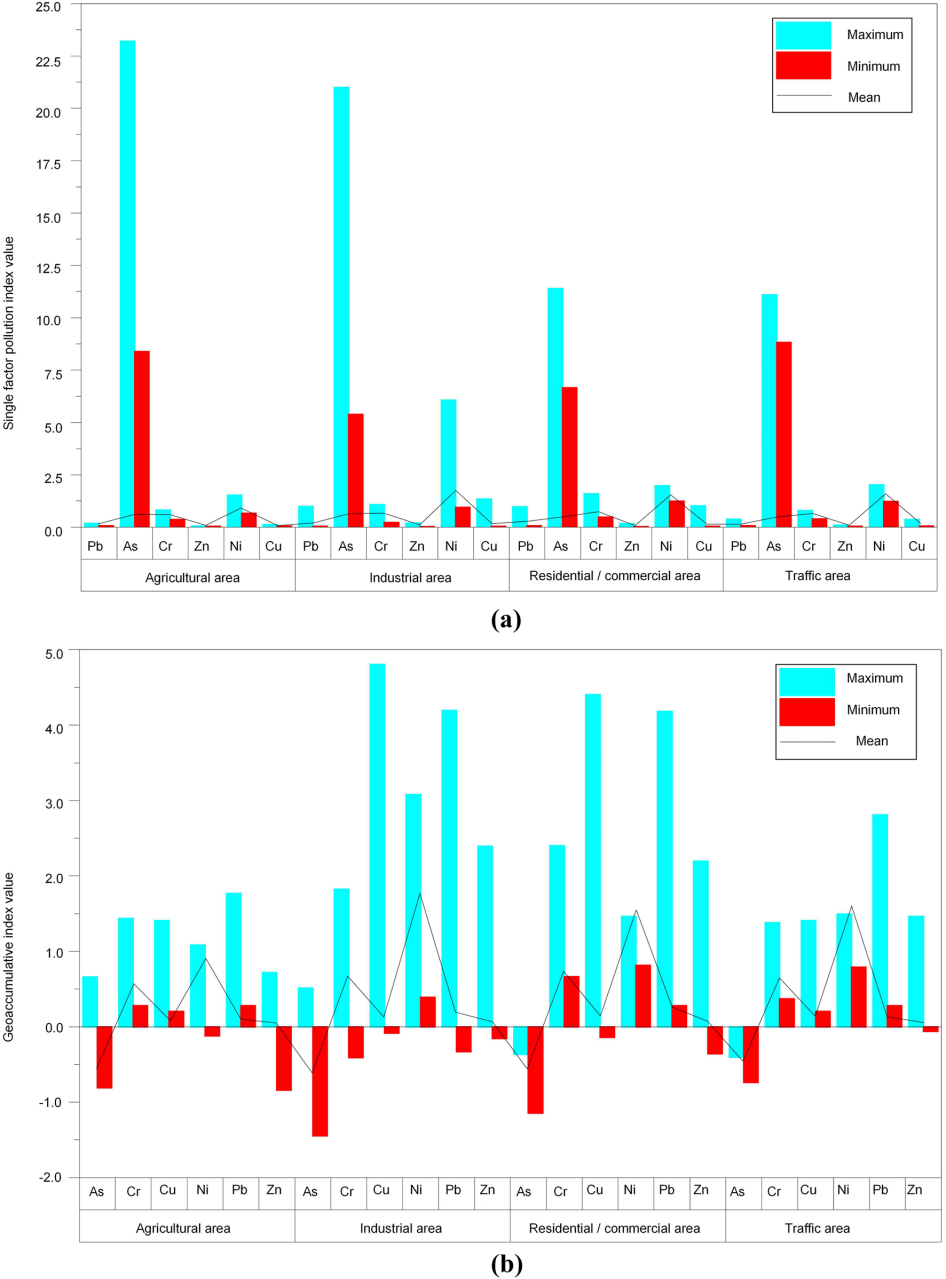
Contamination evaluation of soil heavy metals: **(a)** Single factor index method; **(b)** Geoaccumulation index method; the blue bar represents the maximum contamination, the red bar represents the minimum contamination, and the black line represents the mean contamination.

The experimental data was inserted into formulas (1)–(2), and the effects of different land use types on soil heavy metal pollution were analyzed (Fig. 4, Supplementary Table S4). The results of the single factor index and geo-accumulation index show that Pb and Cr contamination in residential/commercial area > Pb and Cr contamination in industrial area > Pb and Cr contamination in traffic area > Pb and Cr contamination in agricultural area; Ni contamination in industrial area > Ni contamination in traffic area > Ni contamination in residential/commercial area > Ni contamination in agricultural area; and Cu contamination in traffic area > Cu contamination in industrial area > Cu contamination in residential/commercial area > Cu contamination in agricultural area. Due to these differences, it is crucial to analyze the HHRE of heavy metals in contaminated soil.

The second step was to calculate the non-carcinogenic risk of the six heavy metals contained in the 87 soil samples (Supplementary Table S5). The maximum, minimum, and mean concentrations of each heavy metal element in Table 1 have been substituted into formulas (3)–(6). This was done in order to obtain the range of non-carcinogenic risk and carcinogenic risk. In terms of mean values, the non-carcinogenic risk of As was the highest, followed by Pb (Table 5). The risk of As and Pb in industrial, residential/commercial, transportation, and agricultural areas (70.3 and 66.3%, 13.1 and 21.6%, 7.6 and 6.8%, and 9.1 and 5.3%, respectively) indicate that non-carcinogenic risk was greatest in the industrial area. In addition to the aforementioned, the risk of non-carcinogenicity in children is significantly higher than in adults. Moreover, from the data range, the maximum risk of As (5.61) and Pb (9.97) was located in the industrial area, indicating that these brownfield sites pose a high risk for children once they are directly redeveloped into residential and infrastructure lands.

**Table 5:** Non-carcinogenic risk evaluation of soil heavy metals.

Specie of heavy metals	Evaluation index	Non-carcinogenic risk			
		Children		Adults	
		Ranges	Mean	Ranges	Mean
Pb	$HQ_{ois}$	2.89E^-01^–6.66E^+00^	1.25E^+00^	3.40E^-02^–7.84E^-01^	1.47E^-01^
	$HQ_{dcs}$	1.43E^-01^–3.30E^+00^	6.19E^-01^	2.90E^-02^–6.70E^-01^	1.26E^-01^
	$HQ_{pis}$	–	–	0–1.10E^-01^	2.00E^-03^
As	$HQ_{ois}$	1.08E^+00^–4.66E^+00^	2.32E^+00^	1.51E^-01^–6.50E^-01^	3.23E^-01^
	$HQ_{dcs}$	8.10E^-02^–3.48E^-01^	1.73E^-01^	1.60E^-02^–7.00E^-02^	3.50E^-02^
	$HQ_{pis}$	1.40E^-01^–6.03E^-01^	3.00E^-01^	1.40E^-01^–6.03E^-01^	3.00E^-01^
Cr	$HQ_{ois}$	2.29E^-03^–1.61E^-02^	6.71E^-03^	3.21E^-04^–2.25E^-03^	9.40E^-04^
	$HQ_{dcs}$	4.31E^-04^–3.03E^-03^	1.26E^-03^	8.76E^-05^–6.15E^-04^	2.57E^-04^
	$HQ_{pis}$	2.30E^-02^–1.56E^-01^	6.50E^-02^	2.30E^-02^–1.57E^-01^	6.50E^-02^
Zn	$HQ_{ois}$	2.00E^-02^–1.35E^-01^	4.18E^-02^	2.81E^-03^–1.90E^-02^	5.87E^-03^
	$HQ_{dcs}$	4.92E^-05^–3.32E^-04^	1.03E^-04^	1.00E^-05^–6.74E^-05^	2.08E^-05^
	$HQ_{pis}$	–	–	–	–
Ni	$HQ_{ois}$	1.33E^-03^–1.22E^-02^	3.28E^-03^	1.86E^-04^–1.70E^-03^	4.59E^-04^
	$HQ_{dcs}$	6.09E^-03^–5.59E^-02^	1.51E^-02^	1.24E^-03^–1.14E^-02^	3.06E^-03^
	$HQ_{pis}$	1.40E^-02^–1.32E^-01^	3.60E^-02^	1.40E^-02^–1.32E^-01^	3.60E^-02^
Cu	$HQ_{ois}$	3.90E^-02^–1.21E^+00^	1.15E^-01^	5.11E^-03^–1.69E^-01^	1.61E^-02^
	$HQ_{dcs}$	–	–	–	–
	$HQ_{pis}$	–	–	–	–

Thirdly, the carcinogenic risk of As, Cr, and Ni (Table 6). As and Cr was evaluated. It was concluded that these heavy metals pose carcinogenic risks for children and adults, through oral ingestion, dermal contact, and particulate inhalation. More specifically, Cr was shown to have the highest carcinogenic risk of inhalation, with a maximum value of 1.28E−03 and a mean value of 5.35E−04. In general, high-risk areas were primarily located in the industrial area. It is therefore important to remediate health risks caused by heavy metal accumulation in industrial areas.

**Table 6:** Carcinogenic risk evaluation of heavy metals in soil.

Specie of heavy metals	Evaluation index	Carcinogenic risk	
		Ranges	Mean
As	$CR_{ois}$	1.27E^-05^–5.46E^-05^	2.71E^-05^
	$CR_{dcs}$	1.08E^-06^–4.66E^-06^	2.32E^-05^
	$CR_{pis}$	8.83E^-07^–3.80E^-06^	1.89E^-05^
Cr	$CR_{ois}$	4.47E^-05^–3.14E^-04^	1.31E^-04^
	$CR_{dcs}$	5.08E^-06^–3.57E^-05^	1.49E^-05^
	$CR_{pis}$	1.82E^-04^–1.28E^-03^	5.35E^-04^
Ni	$CR_{ois}$	–	–
	$CR_{dcs}$	–	–
	$CR_{pis}$	3.27E^-07^–3.00E^-06^	8.08E^-07^

#### Spatial distribution of HHRE

**Figure 5:**
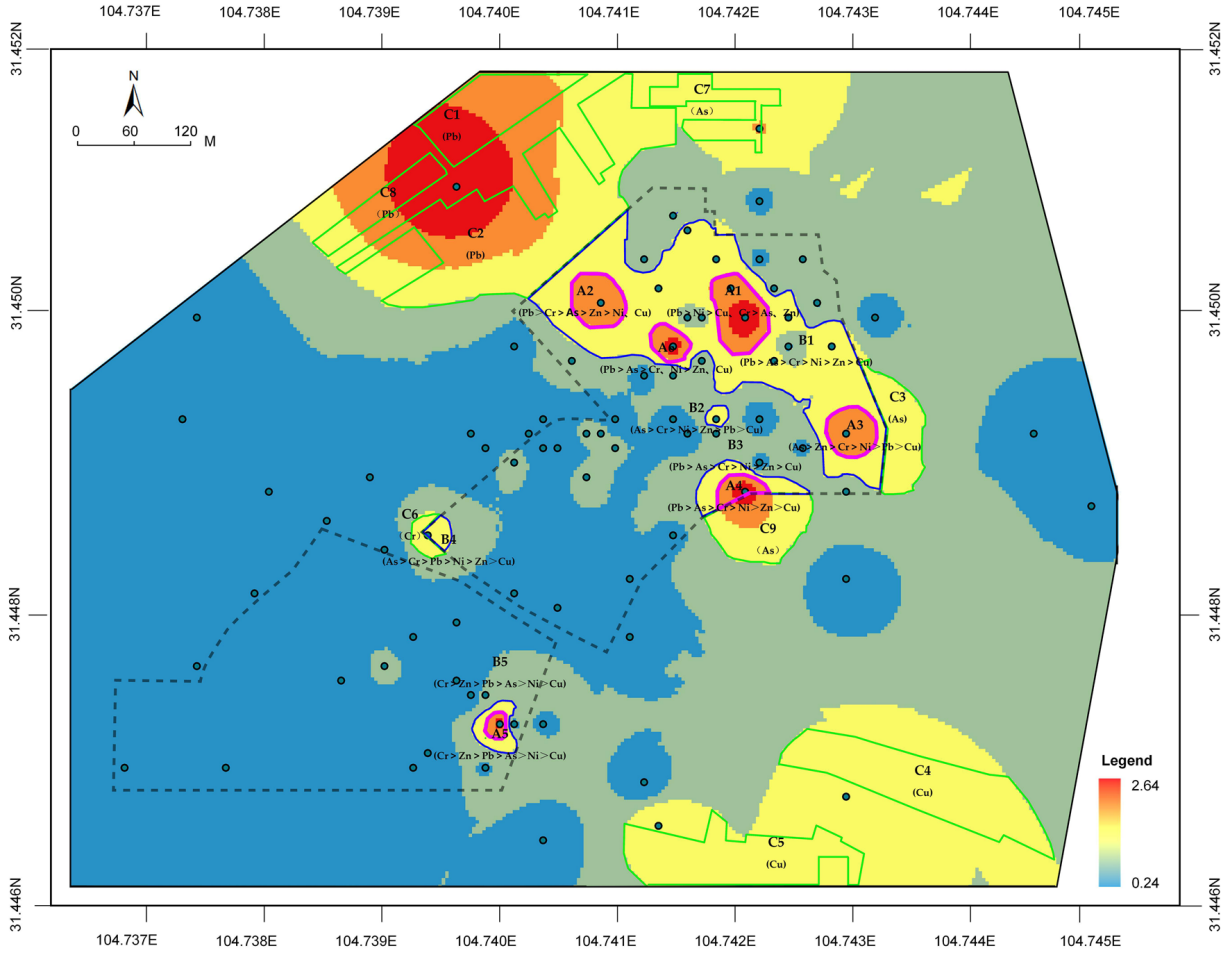
Spatial distribution of non-carcinogenic risk: a color range from blue across yellow to red represented a scale of low to high health risks. (Note: ArcGIS, Version 10.3, ESRI was used to create the map in this figure.)

It is possible that ecological remediation of brownfield sites needs to be planned according to health risk evaluation in order to ensure economic viability. In the present study, the non-carcinogenic risks of Pb, As, Cr, Zn, Ni, and Cu were combined with heavy metal weights. With the command of "weighted sum" in the spatial analysis tool of ArcGIS, the spatial distribution of non-carcinogenic risk caused by six heavy metals on children was vertically superimposed (Fig. 5). According to the construction status of the Mianyang thermal power plant area, apart for the industrial area, other areas are not suitable for large-scale remediation. In the industrial area, plots A1–A5 (pink coil) represent the high-risk area. These primarily envelope the workshops, chemical water treatment area, and coal yard. Furthermore, plots B1–B5 (blue coil) represent the area of medium-risk, concentrated mainly in the warehouses and coal yard. All other spaces in the industrial area were deemed as low risk. In the residential/commercial and transportation area, plots C1–C9 (green coil) represent either a high-risk or medium-risk area. The results indicated that Pb and As are the primary health risk factors in plots A1–A5, while As, Pb, Cr, and Zn are the main health risk factors in plots B1–B5. Lastly, As, Pb, and Cu are primary health risk factors in plots C1–C9.

## Discussion

Health risk evaluation, based on different land use types may assist landscape planners in proposing ecological remediation strategies for brownfield renewal. Through examining the Mianyang thermal power plant area in Sichuan Province, China, this paper has managed to combine the health risk evaluation model with geo-statistics. In turn, this combination has helped determine the different risk levels of areas requiring remediation. The results indicate that statistical tools and ArcGIS are capable of improving the reliability of HHRE with respect to heavy metal pollution in cases of brownfield renewal planning. Thus, the present study helps to propose ecological remediation strategies with additional cost advantages.

Compared to traditional strategies of ecological remediation of contaminated soils, the soil remediation strategies in this paper have conformed to the principles of land resource reuse, site zoning design, and natural succession. Phytoremediation may be used in industrial plots that pose a high health risk, thereby reducing the use of traditional techniques (e.g., soil removal or the sealing of soil surfaces) that interrupt source-receptor pathways^32^. Phytoremediation techniques use wood and herbaceous plants to remove, accumulate, and transfer contaminants^33^. Plant selection in contaminated areas is mainly related to morphological characteristics, the growth environment, heavy metal adsorption capacity, and biomass yield. Therefore, some landscape plants suitable for the local climate conditions were identified. It is expected that they can effectively repair soil contamination (Supplementary Table S6). For A1–A5 plots with high health risks, their industrial activities led to heavy metal contamination in the soil, so these plots can be converted from industrial production to plant growth. For example, Pb and As are the primary health risk factors in plots A1–A5. In order to remediate Pb in the A-type plots, a stratified plant community should be created using a mixture of evergreen trees such as *Ilex chinensis* and *Cinnamomum austrosinense H. T. Chang*, deciduous trees such as *Koelreuteria paniculata*, *Ligustrum lucidum*, and ground cover plants such as *Rhododendron simsii*. *Phragmites communis* and *Pteris vittate* have shown satisfactory remediation effects for As. Furthermore, plots B1–B5, which pose medium health risk, may be redeveloped as entertainment, sports, and other small-scale spaces. Additionally, trees capable of absorbing heavy metals should be planted around these small-scale spaces. The heavy metals As, Pb, Cr, and Zn have been identified as the main health risk factors in plots B1–B5. Phytoremediation of Cr can be achieved using shrubs such as *Pelargonium hortorum* and *Conyza canadensis*. In the case of Zn phytoremediation, *Betula platyphylla*, *Amorpha fruticosa* and *Calendula officinalis* should be used. Lastly, for other spaces with low health risks in the industrial area, multifunctional areas without ecological remediation of the soil can be created, reducing economic costs and improving construction efficiency in turn.

Due to the limitations of urban construction, ecological remediation of contaminated soil in traffic and residential/commercial areas can be achieved through partial renewal. Health risks associated with soil in residential/commercial areas may be due to these areas previously being contaminated industrial and warehouse land. It is suggested that residential/commercial area with high health risks be designed as community gardens with plant landscapes and rain gardens. For example, As, Pb, and Cu have been identified as the health risk factors in plots C1–C9. Cu remediation in plots C4 and C5 may be achieved using shrubs such as *A. fruticosa, L. vicaryi*, coupled with ground cover such as *C. indicum* and *Commelina communis.* Furthermore, soil pollution in the traffic area is the result of accumulation of dust in the soil from automobile exhaust. Therefore, it is necessary to increase the number of plants in the middle and along the edges of roads in these areas. Street trees in Mianyang City, China are mainly evergreen trees, and these trees are required to be high branch point. Therefore, *Cinnamomum Austrosinense H. T. Chang* and *Koelreuteria paniculata* can be selected as street trees for remediation of heavy metal contaminated soil.

Previous studies have mostly used statistical methods to determine soil pollution but have ignored its spatial heterogeneity. This approach may result in excessive remediation and increase treatment costs. This study used ArcGIS to obtain a map of soil health risk instead of the descriptive statistics commonly used in environmental science. The present study was thus able to clarify regional differences of heavy metal pollution. According to the pollution level of the brownfield site, different landscape strategies should be used when soil pollution of heavy metal is remediated, so as to improve the aesthetic value and economic benefit the site.

## Conclusions

Due to rapid industrial development, urban areas have a large number of brownfield resources. However, environmental contamination related to industrial activities may affect residents' health during the process of brownfield renewal. Therefore, in the context of urban renewal, risk evaluation and visual analysis of the heavy metal pollution of soil are necessary to aid brownfield reconstruction and find an ecological remediation strategy. This paper examined the Mianyang thermal power plant area as a case study in order to provide further insight into this issue. Based on the results of a multivariate statistical analysis, spatial distribution analysis, and the HHRE of six heavy metals in soil under four different types of land use, soil with different types of land use was found to pose varying levels of health risk. In the spatial distribution of non-carcinogenic risk, there were five high-risk areas and five medium-risk areas in the industrial area. Moreover, nine high-risk or medium-risk areas were noted in the residential/commercial and transportation areas. This paper has proposed a scheme, a combination of soil experiments, statistical tools, and the ArcGIS tool, that can effectively identify the soil areas that need to be repaired and their degree of pollution. These results can further be used to determine different remediation strategies that may be needed. Additionally, based on the results of the environmental evaluation and methods of landscape planning, the proposed ecological remediation strategy for brownfield renewal has aesthetic value and economic benefit. As China's industry is in a period of transition, more urban brownfield sites are destined to appear in the future. Therefore, research into soil remediation policies that apply to all brownfield renewals is a key area for further study. How to integrate the complete brownfield evaluation, including brownfield identification, brownfield assessment, and brownfield identification based on risk and priority remediation, into the legal system of brownfield governance is a problem worthy of consideration by future researchers. The consistency and comparability of evaluation conclusions can be guided by constructing unified policies for brownfield evaluation.

## Supplementary Information

Supplementary Information.

## Data Availability

Some or all data, models, or code generated or used during the study are available from the corresponding author by request.
