# Supplementary material for: Ecological remediation strategy for urban brownfield renewal in Sichuan Province, China: a health risk evaluation perspective
Source: Sci Rep. 2022 Mar 11;12:4300. doi: 10.1038/s41598-022-08268-z (PMC8917217; doi:10.1038/s41598-022-08268-z)
Supplement: Supplementary file 1 — Supplementary Information. [file 41598_2022_8268_MOESM1_ESM.pdf]

Ecological remediation strategy for urban brownfield renewal in Sichuan Province, China: a health risk evaluation perspective

Weike Zhao[1]; Yuanpei Liao[2]; Shengqiu Zhou[3]; Bo Zhou[*]

[1]College of Architecture and Environment, Sichuan University, Chengdu 610065, China

[2]College of Architecture and Environment, Sichuan University, Chengdu 610065, China

[3]College of Architecture and Environment, Sichuan University, Chengdu 610065, China

*College of Architecture and Environment, Sichuan University, Chengdu 610065, China.

E-mail: zbscuni@163.com; Tel: +86-1898-042-4878

Supplementary information

Supplementary Table S1. Evaluation standard of single factor index method

Grading	P_i	Pollution level
1	$P_i \leq 1$	No pollution
2	$1 < P_i \leq 2$	Mild pollution
3	$2 < P_i \leq 5$	Moderate pollution
4	$P_i > 5$	Severe pollution

Source: Department of Science and Technology Standards, Ministry of Environmental Protection, PRC. *Environmental Protection Standard Book*. China Environmental Science Press (2008)

Supplementary Table S2. Experimental results of heavy metal concentration in soil samples

Sample number	Pb(mg/kg)	As(mg/kg)	Cr(mg/kg)	Zn(mg/kg)	Ni(mg/kg)	Cu(mg/kg)	Longitude (°E)	Latitude (°N)
1	41	11.1	166	172	83	58	104.7407	31.4479
2	30	9.36	168	170	67	48	104.7402	31.4481
3	26	12.2	108	150	63	42	104.7399	31.4491
4	29	11.9	107	136	63	48	104.7404	31.4493
5	53	14.4	186	674	87	74	104.7406	31.4492
6	36	15.2	183	136	88	57	104.7405	31.4493
7	44	13.9	157	126	73	64	104.7402	31.4492
8	46	13.3	156	287	77	64	104.7404	31.4490
9	30	9.41	104	161	78	47	104.7410	31.4486
10	63	12.3	195	174	66	48	104.7407	31.4483
11	44	11.8	191	160	85	41	104.7397	31.4492
12	41	12.0	162	140	93	40	104.74	31.4493
13	31	11.9	147	211	85	30	104.7403	31.4498
14	42	13.2	155	167	83	36	104.7419	31.4492
15	53	15.6	221	241	111	73	104.7413	31.4493
16	40	12.5	153	121	89	35	104.7411	31.4493
17	48	14.1	247	141	102	49	104.7406	31.4494
18	35	9.49	131	141	74	33	104.7408	31.4497
19	36	12.4	164	148	89	37	104.7418	31.4499
20	32	11.1	149	115	84	33	104.7417	31.4498
21	30	14.5	157	161	91	40	104.7412	31.4498
22	95	13.0	191	281	87	62	104.7410	31.4497
23	53	13.7	154	193	84	39	104.7410	31.4494
24	44	13.9	153	156	98	37	104.7413	31.4494
25	40	12.0	156	209	87	37	104.7416	31.4494
26	66	19.2	195	306	99	75	104.7418	31.4501
27	30	10.3	120	128	80	27	104.7409	31.4503
28	76	14.0	257	248	146	73	104.7415	31.4501
29	130	11.7	133	132	79	29	104.7412	31.4501
30	392	14.9	246	401	303	329	104.7411	31.4501
31	91	9.20	144	120	88	30	104.7410	31.4499
32	60	10.9	268	300	85	58	104.7414	31.4503
33	279	12.7	179	273	85	189	104.7413	31.4505
34	162	11.6	186	369	99	801	104.7411	31.4507
35	90	12.1	253	340	111	103	104.7417	31.4503
36	59	17.3	186	250	104	68	104.7420	31.4502
37	85	10.8	165	159	88	45	104.7419	31.4505
38	54	10.9	161	198	89	90	104.7416	31.4505

39	60	12.4	173	214	84	40	104.7410	31.4508
40	51	8.88	131	148	73	29	104.7408	31.4505
41	73	9.63	147	144	86	32	104.7405	31.4502
42	59	12.1	146	134	85	39	104.7421	31.4499
43	209	14.3	211	333	86	52	104.7422	31.4493
44	146	10.7	181	178	90	45	104.7422	31.4489
45	145	21.0	188	502	123	246	104.7415	31.4489
46	77	10.5	179	279	80	162	104.7416	31.4491
47	82	15.4	208	305	95	280	104.7415	31.4489
48	39	8.89	98	168	61	45	104.7390	31.4485
49	47	9.01	166	354	91	222	104.7386	31.4487
50	55	8.82	197	255	101	72	104.7382	31.4489
51	42	11.0	150	156	86	52	104.7381	31.4482
52	26	9.85	140	112	72	30	104.7377	31.4477
53	309	10.1	135	157	78	37	104.7395	31.4480
54	25	9.45	82	138	55	43	104.7392	31.4479
55	50	10.6	181	209	71	55	104.7390	31.4477
56	17	16.4	57	174	52	39	104.7397	31.4475
57	34	7.17	178	131	82	43	104.7397	31.4470
58	64	5.39	157	300	74	58	104.7387	31.4476
59	43	10.3	132	169	62	40	104.7395	31.4476
60	73	23.2	103	184	62	76	104.7398	31.4473
61	34	9.40	141	165	73	34	104.7399	31.4473
62	35	10.0	152	147	70	38	104.7372	31.4470
63	32	14.0	149	121	73	40	104.7379	31.4470
64	41	9.47	139	160	69	39	104.7393	31.4471
65	26	8.38	145	121	74	33	104.7401	31.4465
66	39	8.71	157	150	74	37	104.7409	31.4466
67	34	8.46	161	153	80	35	104.7422	31.4468
68	32	8.68	92	170	48	33	104.7392	31.4470
69	286	8.82	400	587	99	49	104.7401	31.4473
70	56	8.89	154	226	78	34	104.7408	31.4469
71	59	9.64	183	254	81	63	104.7395	31.4510
72	150	9.86	183	137	93	66	104.7377	31.4501
73	34	10.6	125	100	63	26	104.7389	31.4490
74	35	8.64	150	239	74	43	104.7396	31.4493
75	37	10.1	120	133	62	31	104.7401	31.4494
76	179	6.65	344	204	68	33	104.7399	31.4499
77	26	9.02	117	123	62	33	104.7393	31.4486
78	100	11.4	147	542	95	608	104.7422	31.4483
79	44	10.3	168	157	79	51	104.7416	31.4509
80	46	11.1	163	207	80	50	104.7416	31.4514
81	53	7.29	144	177	72	35	104.7424	31.4501
82	389	9.40	170	267	86	57	104.7435	31.4493
83	45	10.1	193	197	83	76	104.7439	31.4488
84	68	8.85	205	211	81	69	104.7376	31.4494
85	49	14.6	208	125	69	64	104.7401	31.4492
86	56	13.4	146	217	47	77	104.7396	31.4475
87	42	13.0	182	118	71	38	104.7399	31.4482

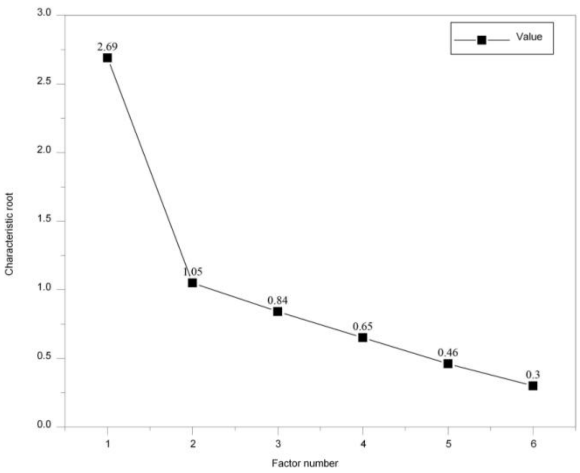

Supplementary Figure S1. Gravel figure of factor analysis: it is used to assist in judging the number of main factors. When the stitches are removed from steep to smooth suddenly, the number of factors corresponding to steep to smooth is the number of main factors. (Note: SPSS 26.0 was used to create this figure.)

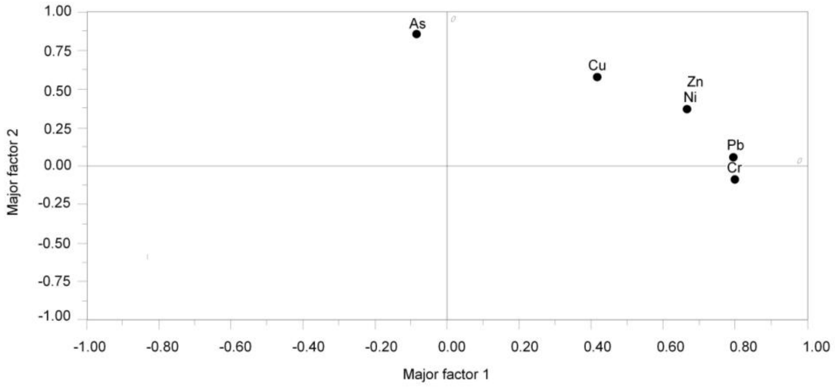

Supplementary Figure S2. Load diagram of Factor analysis: it is used to show the relationship between each factor and the load value (Note: SPSS 26.0 was used to create this figure.)

Supplementary Table S3. Kurtosis and skewness of soil heavy metal concentrations

Name	Kurtosis	Skewness
Pb	8.36	2.86
As	2.86	1.24
Cr	6.53	1.76
Zn	6.06	2.27
Ni	9.92	2.85
Cu	6.31	1.84

Supplementary Table S4. Contamination evaluation of soil by single factor index method and geoaccumulation index method

Land use type		Single factor index（P_i）		Geoaccumulation index（I_{ego}）	
		Ranges	Mean	Ranges	Mean
Industrial area	Pb	0.04-0.98	0.19	-0.33-4.2	1.44

	As	5.39-21	0.62	-1.45-0.52	-0.28
	Cr	0.23-1.07	0.67	-0.41-1.82	1.10
	Zn	0.03-0.19	0.06	-0.16-2.39	0.58
	Ni	0.94-6.06	1.76	0.39-3.08	1.24
	Cu	0.05-1.34	0.13	-0.09-4.8	0.97
Residential/commercial area	Pb	0.07-0.97	0.25	0.28-4.18	1.62
	As	6.65-11.4	0.47	-1.14- -0.37	-0.67
	Cr	0.48-1.6	0.73	0.66-2.4	1.18
	Zn	0.03-0.17	0.07	-0.36-2.19	0.71
	Ni	1.24-1.98	1.56	0.81-1.47	1.11
	Cu	0.04-1.01	0.14	-0.14-4.41	0.76
Traffic area	Pb	0.07-0.38	0.14	0.28-2.81	1.12
	As	8.82-11.1	0.48	-0.74- -0.41	-0.63
	Cr	0.39-0.79	0.64	0.37-1.38	1.05
	Zn	0.04-0.1	0.06	-0.06-1.47	0.54
	Ni	1.22-2.02	1.60	0.79-1.49	1.14
	Cu	0.066-0.37	0.13	0.2-1.41	1.19
Agricultural area	Pb	0.07-0.18	0.11	0.28-1.77	0.89
	As	8.38-23.2	0.57	-0.81-0.66	-0.46
	Cr	0.37-0.82	0.57	0.28-1.44	0.88
	Zn	0.04-0.06	0.05	-0.84-0.72	0.28
	Ni	0.66-1.52	0.90	-0.12-1.08	0.25
	Cu	0.06-0.13	0.08	0.20-1.41	0.57

Supplementary Table S5. HQ and CR evaluation of heavy metals in soil

Supplementary Table S5a. HQ evaluation of Pb

Sample number	HQ_{ois} (Adult)	HQ_{ois} (Child)	HQ_{dcs} (Adult)	HQ_{dcs} (Child)	HQ_{pis} (Adult)
1	5.20E-02	4.42E-01	4.40E-02	2.19E-01	1.00E-03
2	5.80E-02	4.93E-01	5.00E-02	2.44E-01	1.00E-03
3	1.06E-01	9.01E-01	9.10E-02	4.47E-01	2.00E-03
4	7.20E-02	6.12E-01	6.20E-02	3.03E-01	1.00E-03
5	8.80E-02	7.48E-01	7.50E-02	3.71E-01	1.00E-03
6	9.20E-02	7.82E-01	7.90E-02	3.88E-01	1.00E-03
7	6.00E-02	5.10E-01	5.10E-02	2.53E-01	1.00E-03
8	1.26E-01	1.07E+00	1.08E-01	5.31E-01	2.00E-03
9	8.80E-02	7.48E-01	7.50E-02	3.71E-01	1.00E-03
10	8.20E-02	6.97E-01	7.00E-02	3.46E-01	1.00E-03
11	6.20E-02	5.27E-01	5.30E-02	2.61E-01	1.00E-03
12	8.40E-02	7.14E-01	7.20E-02	3.54E-01	1.00E-03
13	1.06E-01	9.01E-01	9.10E-02	4.47E-01	2.00E-03
14	8.00E-02	6.80E-01	6.80E-02	3.37E-01	1.00E-03
15	9.60E-02	8.16E-01	8.20E-02	4.05E-01	1.00E-03

16	7.00E-02	5.95E-01	6.00E-02	2.95E-01	1.00E-03
17	7.20E-02	6.12E-01	6.20E-02	3.03E-01	1.00E-03
18	6.40E-02	5.44E-01	5.50E-02	2.70E-01	1.00E-03
19	6.00E-02	5.10E-01	5.10E-02	2.53E-01	1.00E-03
20	1.90E-01	1.62E+00	1.62E-01	8.01E-01	3.00E-03
21	1.06E-01	9.01E-01	9.10E-02	4.47E-01	2.00E-03
22	8.80E-02	7.48E-01	7.50E-02	3.71E-01	1.00E-03
23	8.00E-02	6.80E-01	6.80E-02	3.37E-01	1.00E-03
24	1.32E-01	1.12E+00	1.13E-01	5.56E-01	2.00E-03
25	6.00E-02	5.10E-01	5.10E-02	2.53E-01	1.00E-03
26	1.52E-01	1.29E+00	1.30E-01	6.41E-01	2.00E-03
27	2.60E-01	2.21E+00	2.22E-01	1.10E+00	4.00E-03
28	7.84E-01	6.66E+00	6.70E-01	3.31E+00	1.10E-02
29	1.82E-01	1.55E+00	1.56E-01	7.67E-01	3.00E-03
30	1.20E-01	1.02E+00	1.03E-01	5.06E-01	2.00E-03
31	5.58E-01	4.74E+00	4.77E-01	2.35E+00	8.00E-03
32	3.24E-01	2.75E+00	2.77E-01	1.37E+00	5.00E-03
33	1.80E-01	1.53E+00	1.54E-01	7.59E-01	3.00E-03
34	1.18E-01	1.00E+00	1.01E-01	4.97E-01	2.00E-03
35	1.70E-01	1.45E+00	1.45E-01	7.17E-01	2.00E-03
36	1.08E-01	9.18E-01	9.20E-02	4.55E-01	2.00E-03
37	1.20E-01	1.02E+00	1.03E-01	5.06E-01	2.00E-03
38	1.02E-01	8.67E-01	8.70E-02	4.30E-01	1.00E-03
39	1.46E-01	1.24E+00	1.25E-01	6.15E-01	2.00E-03
40	1.18E-01	1.00E+00	1.01E-01	4.97E-01	2.00E-03
41	4.18E-01	3.55E+00	3.57E-01	1.76E+00	6.00E-03
42	2.92E-01	2.48E+00	2.50E-01	1.23E+00	4.00E-03
43	2.90E-01	2.47E+00	2.48E-01	1.22E+00	4.00E-03
44	1.54E-01	1.31E+00	1.32E-01	6.49E-01	2.00E-03
45	1.64E-01	1.39E+00	1.40E-01	6.91E-01	2.00E-03
46	8.40E-02	7.14E-01	7.20E-02	3.54E-01	1.00E-03
47	6.18E-01	5.25E+00	5.28E-01	2.61E+00	9.00E-03
48	5.00E-02	4.25E-01	4.30E-02	2.11E-01	1.00E-03
49	1.00E-01	8.50E-01	8.60E-02	4.22E-01	1.00E-03
50	3.40E-02	2.89E-01	2.90E-02	1.43E-01	0.00E+00
51	6.80E-02	5.78E-01	5.80E-02	2.87E-01	1.00E-03
52	1.28E-01	1.09E+00	1.09E-01	5.40E-01	2.00E-03
53	8.60E-02	7.31E-01	7.40E-02	3.62E-01	1.00E-03
54	8.20E-02	6.97E-01	7.00E-02	3.46E-01	1.00E-03
55	9.80E-02	8.33E-01	8.40E-02	4.13E-01	1.00E-03
56	1.12E-01	9.52E-01	9.60E-02	4.72E-01	2.00E-03
57	8.40E-02	7.14E-01	7.20E-02	3.54E-01	1.00E-03
58	5.20E-02	4.42E-01	4.40E-02	2.19E-01	1.00E-03
59	6.80E-02	5.78E-01	5.80E-02	2.87E-01	1.00E-03
60	5.72E-01	4.86E+00	4.89E-01	2.41E+00	8.00E-03
61	1.12E-01	9.52E-01	9.60E-02	4.72E-01	2.00E-03

62	1.18E-01	1.00E+00	1.01E-01	4.97E-01	2.00E-03
63	6.80E-02	5.78E-01	5.80E-02	2.87E-01	1.00E-03
64	7.00E-02	5.95E-01	6.00E-02	2.95E-01	1.00E-03
65	7.40E-02	6.29E-01	6.30E-02	3.12E-01	1.00E-03
66	3.58E-01	3.04E+00	3.06E-01	1.51E+00	5.00E-03
67	2.00E-01	1.70E+00	1.71E-01	8.43E-01	3.00E-03
68	8.80E-02	7.48E-01	7.50E-02	3.71E-01	1.00E-03
69	9.20E-02	7.82E-01	7.90E-02	3.88E-01	1.00E-03
70	1.06E-01	9.01E-01	9.10E-02	4.47E-01	2.00E-03
71	7.78E-01	6.61E+00	6.65E-01	3.28E+00	1.10E-02
72	8.20E-02	6.97E-01	7.00E-02	3.46E-01	1.00E-03
73	6.00E-02	5.10E-01	5.10E-02	2.53E-01	1.00E-03
74	7.80E-02	6.63E-01	6.70E-02	3.29E-01	1.00E-03
75	9.40E-02	7.99E-01	8.00E-02	3.96E-01	1.00E-03
76	1.10E-01	9.35E-01	9.40E-02	4.64E-01	2.00E-03
77	3.00E-01	2.55E+00	2.57E-01	1.27E+00	4.00E-03
78	5.20E-02	4.42E-01	4.40E-02	2.19E-01	1.00E-03
79	9.00E-02	7.65E-01	7.70E-02	3.79E-01	1.00E-03
80	1.46E-01	1.24E+00	1.25E-01	6.15E-01	2.00E-03
81	6.80E-02	5.78E-01	5.80E-02	2.87E-01	1.00E-03
82	7.00E-02	5.95E-01	6.00E-02	2.95E-01	1.00E-03
83	6.40E-02	5.44E-01	5.50E-02	2.70E-01	1.00E-03
84	5.20E-02	4.42E-01	4.40E-02	2.19E-01	1.00E-03
85	7.80E-02	6.63E-01	6.70E-02	3.29E-01	1.00E-03
86	6.40E-02	5.44E-01	5.50E-02	2.70E-01	1.00E-03
87	1.36E-01	1.16E+00	1.16E-01	5.73E-01	2.00E-03

Supplementary Table S5b. HQ and CR evaluation of As

Sample number	HQ_{ois} (Adult)	HQ_{ois} (Child)	HQ_{dcs} (Adult)	HQ_{dcs} (Child)	HQ_{pis} (Adult)	HQ_{pis} (Child)	CR_{ois}	CR_{dcs}	CR_{pis}
1	3.42E-01	2.45E+00	3.70E-02	1.83E-01	3.17E-01	3.17E-01	2.87E-05	2.45E-06	2.00E-06
2	3.33E-01	2.39E+00	3.60E-02	1.79E-01	3.09E-01	3.09E-01	2.80E-05	2.39E-06	1.95E-06
3	4.03E-01	2.89E+00	4.30E-02	2.16E-01	3.74E-01	3.74E-01	3.39E-05	2.89E-06	2.36E-06
4	4.26E-01	3.06E+00	4.60E-02	2.28E-01	3.95E-01	3.95E-01	3.58E-05	3.05E-06	2.49E-06
5	3.89E-01	2.79E+00	4.20E-02	2.09E-01	3.61E-01	3.61E-01	3.27E-05	2.79E-06	2.28E-06
6	3.72E-01	2.67E+00	4.00E-02	2.00E-01	3.46E-01	3.46E-01	3.13E-05	2.67E-06	2.18E-06
7	2.63E-01	1.89E+00	2.80E-02	1.41E-01	2.45E-01	2.45E-01	2.21E-05	1.89E-06	1.54E-06
8	3.44E-01	2.47E+00	3.70E-02	1.85E-01	3.20E-01	3.20E-01	2.89E-05	2.47E-06	2.02E-06
9	3.30E-01	2.37E+00	3.50E-02	1.77E-01	3.07E-01	3.07E-01	2.78E-05	2.37E-06	1.93E-06
10	3.36E-01	2.41E+00	3.60E-02	1.80E-01	3.12E-01	3.12E-01	2.82E-05	2.41E-06	1.97E-06
11	3.33E-01	2.39E+00	3.60E-02	1.79E-01	3.09E-01	3.09E-01	2.80E-05	2.39E-06	1.95E-06
12	3.70E-01	2.65E+00	4.00E-02	1.98E-01	3.43E-01	3.43E-01	3.11E-05	2.65E-06	2.16E-06
13	4.37E-01	3.14E+00	4.70E-02	2.34E-01	4.06E-01	4.06E-01	3.67E-05	3.13E-06	2.56E-06
14	3.50E-01	2.51E+00	3.80E-02	1.88E-01	3.25E-01	3.25E-01	2.94E-05	2.51E-06	2.05E-06
15	3.95E-01	2.83E+00	4.20E-02	2.12E-01	3.67E-01	3.67E-01	3.32E-05	2.83E-06	2.31E-06

16	2.66E-01	1.91E+00	2.80E-02	1.42E-01	2.47E-01	2.47E-01	2.23E-05	1.90E-06	1.56E-06
17	3.47E-01	2.49E+00	3.70E-02	1.86E-01	3.22E-01	3.22E-01	2.92E-05	2.49E-06	2.03E-06
18	3.11E-01	2.23E+00	3.30E-02	1.67E-01	2.89E-01	2.89E-01	2.61E-05	2.23E-06	1.82E-06
19	4.06E-01	2.92E+00	4.40E-02	2.18E-01	3.77E-01	3.77E-01	3.41E-05	2.91E-06	2.38E-06
20	3.64E-01	2.61E+00	3.90E-02	1.95E-01	3.38E-01	3.38E-01	3.06E-05	2.61E-06	2.13E-06
21	3.84E-01	2.75E+00	4.10E-02	2.06E-01	3.56E-01	3.56E-01	3.22E-05	2.75E-06	2.25E-06
22	3.89E-01	2.79E+00	4.20E-02	2.09E-01	3.61E-01	3.61E-01	3.27E-05	2.79E-06	2.28E-06
23	3.36E-01	2.41E+00	3.60E-02	1.80E-01	3.12E-01	3.12E-01	2.82E-05	2.41E-06	1.97E-06
24	5.38E-01	3.86E+00	5.80E-02	2.88E-01	4.99E-01	4.99E-01	4.52E-05	3.85E-06	3.15E-06
25	2.88E-01	2.07E+00	3.10E-02	1.55E-01	2.68E-01	2.68E-01	2.42E-05	2.07E-06	1.69E-06
26	3.92E-01	2.81E+00	4.20E-02	2.10E-01	3.64E-01	3.64E-01	3.29E-05	2.81E-06	2.29E-06
27	3.28E-01	2.35E+00	3.50E-02	1.76E-01	3.04E-01	3.04E-01	2.75E-05	2.35E-06	1.92E-06
28	4.17E-01	3.00E+00	4.50E-02	2.24E-01	3.87E-01	3.87E-01	3.50E-05	2.99E-06	2.44E-06
29	2.58E-01	1.85E+00	2.80E-02	1.38E-01	2.39E-01	2.39E-01	2.16E-05	1.85E-06	1.51E-06
30	3.05E-01	2.19E+00	3.30E-02	1.64E-01	2.83E-01	2.83E-01	2.56E-05	2.19E-06	1.79E-06
31	3.56E-01	2.55E+00	3.80E-02	1.91E-01	3.30E-01	3.30E-01	2.99E-05	2.55E-06	2.08E-06
32	3.25E-01	2.33E+00	3.50E-02	1.74E-01	3.02E-01	3.02E-01	2.73E-05	2.33E-06	1.90E-06
33	3.39E-01	2.43E+00	3.60E-02	1.82E-01	3.15E-01	3.15E-01	2.85E-05	2.43E-06	1.98E-06
34	4.84E-01	3.48E+00	5.20E-02	2.60E-01	4.50E-01	4.50E-01	4.07E-05	3.47E-06	2.84E-06
35	3.02E-01	2.17E+00	3.20E-02	1.62E-01	2.81E-01	2.81E-01	2.54E-05	2.17E-06	1.77E-06
36	3.05E-01	2.19E+00	3.30E-02	1.64E-01	2.83E-01	2.83E-01	2.56E-05	2.19E-06	1.79E-06
37	3.47E-01	2.49E+00	3.70E-02	1.86E-01	3.22E-01	3.22E-01	2.92E-05	2.49E-06	2.03E-06
38	2.49E-01	1.79E+00	2.70E-02	1.33E-01	2.31E-01	2.31E-01	2.09E-05	1.78E-06	1.46E-06
39	2.70E-01	1.94E+00	2.90E-02	1.44E-01	2.50E-01	2.50E-01	2.27E-05	1.93E-06	1.58E-06
40	3.39E-01	2.43E+00	3.60E-02	1.82E-01	3.15E-01	3.15E-01	2.85E-05	2.43E-06	1.98E-06
41	4.00E-01	2.87E+00	4.30E-02	2.15E-01	3.72E-01	3.72E-01	3.36E-05	2.87E-06	2.34E-06
42	3.00E-01	2.15E+00	3.20E-02	1.61E-01	2.78E-01	2.78E-01	2.52E-05	2.15E-06	1.75E-06
43	5.88E-01	4.22E+00	6.30E-02	3.15E-01	5.46E-01	5.46E-01	4.94E-05	4.21E-06	3.44E-06
44	2.94E-01	2.11E+00	3.20E-02	1.58E-01	2.73E-01	2.73E-01	2.47E-05	2.11E-06	1.72E-06
45	4.31E-01	3.10E+00	4.60E-02	2.31E-01	4.00E-01	4.00E-01	3.62E-05	3.09E-06	2.52E-06
46	3.08E-01	2.21E+00	3.30E-02	1.65E-01	2.86E-01	2.86E-01	2.59E-05	2.21E-06	1.80E-06
47	2.83E-01	2.03E+00	3.00E-02	1.52E-01	2.63E-01	2.63E-01	2.38E-05	2.03E-06	1.66E-06
48	2.65E-01	1.90E+00	2.80E-02	1.42E-01	2.46E-01	2.46E-01	2.22E-05	1.90E-06	1.55E-06
49	2.97E-01	2.13E+00	3.20E-02	1.59E-01	2.76E-01	2.76E-01	2.49E-05	2.13E-06	1.74E-06
50	4.59E-01	3.30E+00	4.90E-02	2.46E-01	4.26E-01	4.26E-01	3.86E-05	3.29E-06	2.69E-06
51	2.01E-01	1.44E+00	2.20E-02	1.08E-01	1.86E-01	1.86E-01	1.69E-05	1.44E-06	1.18E-06
52	1.51E-01	1.08E+00	1.60E-02	8.10E-02	1.40E-01	1.40E-01	1.27E-05	1.08E-06	8.83E-07
53	2.88E-01	2.07E+00	3.10E-02	1.55E-01	2.68E-01	2.68E-01	2.42E-05	2.07E-06	1.69E-06
54	2.65E-01	1.90E+00	2.80E-02	1.42E-01	2.46E-01	2.46E-01	2.23E-05	1.90E-06	1.55E-06
55	4.09E-01	2.94E+00	4.40E-02	2.19E-01	3.80E-01	3.80E-01	3.43E-05	2.93E-06	2.39E-06
56	3.75E-01	2.69E+00	4.00E-02	2.01E-01	3.48E-01	3.48E-01	3.15E-05	2.69E-06	2.20E-06
57	3.64E-01	2.61E+00	3.90E-02	1.95E-01	3.38E-01	3.38E-01	3.06E-05	2.61E-06	2.13E-06
58	2.76E-01	1.98E+00	3.00E-02	1.48E-01	2.56E-01	2.56E-01	2.32E-05	1.98E-06	1.61E-06
59	2.37E-01	1.70E+00	2.50E-02	1.27E-01	2.20E-01	2.20E-01	1.99E-05	1.70E-06	1.39E-06

60	2.47E-01	1.77E+00	2.60E-02	1.32E-01	2.29E-01	2.29E-01	2.07E-05	1.77E-06	1.45E-06
61	2.49E-01	1.79E+00	2.70E-02	1.33E-01	2.31E-01	2.31E-01	2.09E-05	1.78E-06	1.46E-06
62	2.70E-01	1.94E+00	2.90E-02	1.45E-01	2.51E-01	2.51E-01	2.27E-05	1.93E-06	1.58E-06
63	2.97E-01	2.13E+00	3.20E-02	1.59E-01	2.76E-01	2.76E-01	2.49E-05	2.13E-06	1.74E-06
64	2.42E-01	1.74E+00	2.60E-02	1.30E-01	2.25E-01	2.25E-01	2.03E-05	1.73E-06	1.42E-06
65	2.83E-01	2.03E+00	3.00E-02	1.52E-01	2.63E-01	2.63E-01	2.38E-05	2.03E-06	1.66E-06
66	1.86E-01	1.34E+00	2.00E-02	1.00E-01	1.73E-01	1.73E-01	1.56E-05	1.33E-06	1.09E-06
67	3.19E-01	2.29E+00	3.40E-02	1.71E-01	2.96E-01	2.96E-01	2.68E-05	2.29E-06	1.87E-06
68	2.88E-01	2.07E+00	3.10E-02	1.55E-01	2.68E-01	2.68E-01	2.42E-05	2.07E-06	1.69E-06
69	3.11E-01	2.23E+00	3.30E-02	1.67E-01	2.89E-01	2.89E-01	2.61E-05	2.23E-06	1.82E-06
70	2.04E-01	1.47E+00	2.20E-02	1.09E-01	1.90E-01	1.90E-01	1.72E-05	1.46E-06	1.20E-06
71	2.63E-01	1.89E+00	2.80E-02	1.41E-01	2.44E-01	2.44E-01	2.21E-05	1.89E-06	1.54E-06
72	3.11E-01	2.23E+00	3.30E-02	1.67E-01	2.89E-01	2.89E-01	2.61E-05	2.23E-06	1.82E-06
73	2.62E-01	1.88E+00	2.80E-02	1.40E-01	2.43E-01	2.43E-01	2.20E-05	1.88E-06	1.53E-06
74	2.49E-01	1.79E+00	2.70E-02	1.33E-01	2.31E-01	2.31E-01	2.09E-05	1.78E-06	1.46E-06
75	2.52E-01	1.81E+00	2.70E-02	1.35E-01	2.34E-01	2.34E-01	2.12E-05	1.81E-06	1.48E-06
76	2.47E-01	1.77E+00	2.60E-02	1.32E-01	2.29E-01	2.29E-01	2.07E-05	1.77E-06	1.45E-06
77	2.76E-01	1.98E+00	3.00E-02	1.48E-01	2.56E-01	2.56E-01	2.32E-05	1.98E-06	1.62E-06
78	2.53E-01	1.81E+00	2.70E-02	1.35E-01	2.35E-01	2.35E-01	2.12E-05	1.81E-06	1.48E-06
79	2.83E-01	2.03E+00	3.00E-02	1.52E-01	2.63E-01	2.63E-01	2.38E-05	2.03E-06	1.66E-06
80	6.50E-01	4.66E+00	7.00E-02	3.48E-01	6.03E-01	6.03E-01	5.46E-05	4.66E-06	3.80E-06
81	2.63E-01	1.89E+00	2.80E-02	1.41E-01	2.44E-01	2.44E-01	2.21E-05	1.89E-06	1.54E-06
82	2.80E-01	2.01E+00	3.00E-02	1.50E-01	2.60E-01	2.60E-01	2.35E-05	2.01E-06	1.64E-06
83	3.92E-01	2.81E+00	4.20E-02	2.10E-01	3.64E-01	3.64E-01	3.29E-05	2.81E-06	2.29E-06
84	2.35E-01	1.68E+00	2.50E-02	1.26E-01	2.18E-01	2.18E-01	1.97E-05	1.68E-06	1.37E-06
85	2.44E-01	1.75E+00	2.60E-02	1.31E-01	2.26E-01	2.26E-01	2.05E-05	1.75E-06	1.43E-06
86	2.43E-01	1.75E+00	2.60E-02	1.30E-01	2.26E-01	2.26E-01	2.04E-05	1.74E-06	1.42E-06
87	2.48E-01	1.78E+00	2.70E-02	1.33E-01	2.30E-01	2.30E-01	2.08E-05	1.78E-06	1.45E-06

* CR_{ois} ： Carcinogenic risk from oral ingestion of soil; CR_{dcs} ： Carcinogenic risk from dermal contact of soil; CR_{pis} ： Carcinogenic risk from inhalation of soil.

Supplementary Table S5c. HQ and CR evaluation of Cr

Sample number	HQ_{ois} (Adult)	HQ_{ois} (Child)	HQ_{dcs} (Adult)	HQ_{dcs} (Child)	HQ_{pis} (Adult)	HQ_{pis} (Child)	CR_{ois}	CR_{dcs}	CR_{pis}
1	6.08E-04	4.34E-03	1.66E-04	8.17E-04	4.23E-02	4.22E-02	8.47E-05	9.63E-06	3.46E-04
2	6.02E-04	4.30E-03	1.65E-04	8.10E-04	4.19E-02	4.18E-02	8.39E-05	9.54E-06	3.43E-04
3	1.05E-03	7.48E-03	2.86E-04	1.41E-03	7.28E-02	7.27E-02	1.46E-04	1.66E-05	5.95E-04
4	1.03E-03	7.36E-03	2.81E-04	1.39E-03	7.16E-02	7.16E-02	1.43E-04	1.63E-05	5.86E-04
5	8.83E-04	6.31E-03	2.41E-04	1.19E-03	6.14E-02	6.14E-02	1.23E-04	1.40E-05	5.03E-04
6	8.78E-04	6.27E-03	2.40E-04	1.18E-03	6.11E-02	6.10E-02	1.22E-04	1.39E-05	4.99E-04
7	5.85E-04	4.18E-03	1.60E-04	7.87E-04	4.07E-02	4.07E-02	8.15E-05	9.27E-06	3.33E-04
8	1.10E-03	7.84E-03	3.00E-04	1.48E-03	7.63E-02	7.63E-02	1.53E-04	1.74E-05	6.24E-04

9	1.07E-03	7.68E-03	2.94E-04	1.45E-03	7.48E-02	7.47E-02	1.50E-04	1.70E-05	6.11E-04
10	9.12E-04	6.51E-03	2.49E-04	1.23E-03	6.34E-02	6.33E-02	1.27E-04	1.44E-05	5.19E-04
11	8.27E-04	5.91E-03	2.26E-04	1.11E-03	5.75E-02	5.75E-02	1.15E-04	1.31E-05	4.71E-04
12	8.72E-04	6.23E-03	2.38E-04	1.17E-03	6.07E-02	6.06E-02	1.22E-04	1.38E-05	4.96E-04
13	1.24E-03	8.88E-03	3.40E-04	1.67E-03	8.65E-02	8.64E-02	1.73E-04	1.97E-05	7.07E-04
14	8.61E-04	6.15E-03	2.35E-04	1.16E-03	5.99E-02	5.98E-02	1.20E-04	1.36E-05	4.90E-04
15	1.39E-03	9.93E-03	3.80E-04	1.87E-03	9.67E-02	9.66E-02	1.94E-04	2.20E-05	7.91E-04
16	7.37E-04	5.27E-03	2.01E-04	9.92E-04	5.13E-02	5.12E-02	1.03E-04	1.17E-05	4.19E-04
17	9.23E-04	6.59E-03	2.52E-04	1.24E-03	6.42E-02	6.41E-02	1.29E-04	1.46E-05	5.25E-04
18	8.38E-04	5.99E-03	2.29E-04	1.13E-03	5.83E-02	5.83E-02	1.17E-04	1.33E-05	4.77E-04
19	8.83E-04	6.31E-03	2.41E-04	1.19E-03	6.14E-02	6.14E-02	1.23E-04	1.40E-05	5.03E-04
20	1.07E-03	7.68E-03	2.94E-04	1.45E-03	7.48E-02	7.47E-02	1.50E-04	1.70E-05	6.11E-04
21	8.67E-04	6.19E-03	2.37E-04	1.17E-03	6.03E-02	6.02E-02	1.21E-04	1.37E-05	4.93E-04
22	8.61E-04	6.15E-03	2.35E-04	1.16E-03	5.99E-02	5.98E-02	1.20E-04	1.36E-05	4.90E-04
23	8.78E-04	6.27E-03	2.40E-04	1.18E-03	6.11E-02	6.10E-02	1.22E-04	1.39E-05	4.99E-04
24	1.10E-03	7.84E-03	3.00E-04	1.48E-03	7.63E-02	7.63E-02	1.53E-04	1.74E-05	6.24E-04
25	6.75E-04	4.82E-03	1.85E-04	9.08E-04	4.70E-02	4.69E-02	9.41E-05	1.07E-05	3.84E-04
26	1.45E-03	1.03E-02	3.95E-04	1.95E-03	1.01E-01	1.00E-01	2.01E-04	2.29E-05	8.23E-04
27	7.48E-04	5.35E-03	2.05E-04	1.01E-03	5.21E-02	5.20E-02	1.04E-04	1.19E-05	4.26E-04
28	1.38E-03	9.89E-03	3.78E-04	1.86E-03	9.63E-02	9.62E-02	1.93E-04	2.19E-05	7.88E-04
29	8.10E-04	5.79E-03	2.21E-04	1.09E-03	5.64E-02	5.63E-02	1.13E-04	1.28E-05	4.61E-04
30	1.51E-03	1.08E-02	4.12E-04	2.03E-03	1.05E-01	1.05E-01	2.10E-04	2.39E-05	8.58E-04
31	1.01E-03	7.20E-03	2.75E-04	1.35E-03	7.01E-02	7.00E-02	1.40E-04	1.60E-05	5.73E-04
32	1.05E-03	7.48E-03	2.86E-04	1.41E-03	7.28E-02	7.27E-02	1.46E-04	1.66E-05	5.95E-04
33	1.42E-03	1.02E-02	3.89E-04	1.92E-03	9.90E-02	9.89E-02	1.98E-04	2.26E-05	8.10E-04
34	1.05E-03	7.48E-03	2.86E-04	1.41E-03	7.28E-02	7.27E-02	1.46E-04	1.66E-05	5.95E-04
35	9.28E-04	6.63E-03	2.54E-04	1.25E-03	6.46E-02	6.45E-02	1.29E-04	1.47E-05	5.28E-04
36	9.06E-04	6.47E-03	2.48E-04	1.22E-03	6.30E-02	6.30E-02	1.26E-04	1.44E-05	5.15E-04
37	9.73E-04	6.95E-03	2.66E-04	1.31E-03	6.77E-02	6.76E-02	1.36E-04	1.54E-05	5.54E-04
38	7.37E-04	5.27E-03	2.01E-04	9.92E-04	5.13E-02	5.12E-02	1.03E-04	1.17E-05	4.19E-04
39	8.27E-04	5.91E-03	2.26E-04	1.11E-03	5.75E-02	5.75E-02	1.15E-04	1.31E-05	4.71E-04
40	8.21E-04	5.87E-03	2.25E-04	1.11E-03	5.71E-02	5.71E-02	1.14E-04	1.30E-05	4.67E-04
41	1.19E-03	8.48E-03	3.24E-04	1.60E-03	8.26E-02	8.25E-02	1.65E-04	1.88E-05	6.75E-04
42	1.02E-03	7.28E-03	2.78E-04	1.37E-03	7.08E-02	7.08E-02	1.42E-04	1.61E-05	5.79E-04
43	1.06E-03	7.56E-03	2.89E-04	1.42E-03	7.36E-02	7.35E-02	1.47E-04	1.68E-05	6.02E-04
44	1.01E-03	7.20E-03	2.75E-04	1.35E-03	7.01E-02	7.00E-02	1.40E-04	1.60E-05	5.73E-04
45	1.17E-03	8.36E-03	3.20E-04	1.57E-03	8.14E-02	8.13E-02	1.63E-04	1.85E-05	6.66E-04
46	8.44E-04	6.03E-03	2.31E-04	1.14E-03	5.87E-02	5.87E-02	1.18E-04	1.34E-05	4.80E-04
47	7.60E-04	5.43E-03	2.08E-04	1.02E-03	5.28E-02	5.28E-02	1.06E-04	1.20E-05	4.32E-04
48	4.61E-04	3.30E-03	1.26E-04	6.21E-04	3.21E-02	3.21E-02	6.43E-05	7.31E-06	2.63E-04
49	1.02E-03	7.28E-03	2.78E-04	1.37E-03	7.08E-02	7.08E-02	1.42E-04	1.61E-05	5.79E-04
50	3.21E-04	2.29E-03	8.76E-05	4.31E-04	2.23E-02	2.23E-02	4.47E-05	5.08E-06	1.82E-04
51	1.00E-03	7.16E-03	2.74E-04	1.35E-03	6.97E-02	6.96E-02	1.40E-04	1.59E-05	5.70E-04
52	8.83E-04	6.31E-03	2.41E-04	1.19E-03	6.14E-02	6.14E-02	1.23E-04	1.40E-05	5.03E-04

53	7.43E-04	5.31E-03	2.03E-04	9.99E-04	5.17E-02	5.16E-02	1.03E-04	1.18E-05	4.23E-04
54	7.82E-04	5.59E-03	2.14E-04	1.05E-03	5.44E-02	5.44E-02	1.09E-04	1.24E-05	4.45E-04
55	1.17E-03	8.36E-03	3.20E-04	1.57E-03	8.14E-02	8.13E-02	1.63E-04	1.85E-05	6.66E-04
56	8.21E-04	5.87E-03	2.25E-04	1.11E-03	5.71E-02	5.71E-02	1.14E-04	1.30E-05	4.67E-04
57	1.02E-03	7.32E-03	2.80E-04	1.38E-03	7.12E-02	7.12E-02	1.43E-04	1.62E-05	5.83E-04
58	7.88E-04	5.63E-03	2.15E-04	1.06E-03	5.48E-02	5.47E-02	1.10E-04	1.25E-05	4.48E-04
59	9.06E-04	6.47E-03	2.48E-04	1.22E-03	6.30E-02	6.30E-02	1.26E-04	1.44E-05	5.15E-04
60	2.25E-03	1.61E-02	6.15E-04	3.03E-03	1.57E-01	1.56E-01	3.14E-04	3.57E-05	1.28E-03
61	8.67E-04	6.19E-03	2.37E-04	1.17E-03	6.03E-02	6.02E-02	1.21E-04	1.37E-05	4.93E-04
62	1.03E-03	7.36E-03	2.81E-04	1.39E-03	7.16E-02	7.16E-02	1.43E-04	1.63E-05	5.86E-04
63	7.03E-04	5.03E-03	1.92E-04	9.46E-04	4.89E-02	4.89E-02	9.80E-05	1.11E-05	4.00E-04
64	8.44E-04	6.03E-03	2.31E-04	1.14E-03	5.87E-02	5.87E-02	1.18E-04	1.34E-05	4.80E-04
65	6.75E-04	4.82E-03	1.85E-04	9.08E-04	4.70E-02	4.69E-02	9.41E-05	1.07E-05	3.84E-04
66	1.94E-03	1.38E-02	5.29E-04	2.60E-03	1.35E-01	1.35E-01	2.70E-04	3.07E-05	1.10E-03
67	8.27E-04	5.91E-03	2.26E-04	1.11E-03	5.75E-02	5.75E-02	1.15E-04	1.31E-05	4.71E-04
68	9.45E-04	6.75E-03	2.58E-04	1.27E-03	6.58E-02	6.57E-02	1.32E-04	1.50E-05	5.38E-04
69	9.17E-04	6.55E-03	2.51E-04	1.23E-03	6.38E-02	6.37E-02	1.28E-04	1.45E-05	5.22E-04
70	8.10E-04	5.79E-03	2.21E-04	1.09E-03	5.64E-02	5.63E-02	1.13E-04	1.28E-05	4.61E-04
71	9.57E-04	6.83E-03	2.61E-04	1.29E-03	6.65E-02	6.65E-02	1.33E-04	1.52E-05	5.44E-04
72	9.34E-04	6.67E-03	2.55E-04	1.26E-03	6.50E-02	6.49E-02	1.30E-04	1.48E-05	5.31E-04
73	9.45E-04	6.75E-03	2.58E-04	1.27E-03	6.58E-02	6.57E-02	1.32E-04	1.50E-05	5.38E-04
74	5.51E-04	3.94E-03	1.51E-04	7.42E-04	3.84E-02	3.83E-02	7.68E-05	8.74E-06	3.14E-04
75	9.34E-04	6.67E-03	2.55E-04	1.26E-03	6.50E-02	6.49E-02	1.30E-04	1.48E-05	5.31E-04
76	1.11E-03	7.92E-03	3.03E-04	1.49E-03	7.71E-02	7.70E-02	1.54E-04	1.76E-05	6.31E-04
77	1.03E-03	7.36E-03	2.81E-04	1.39E-03	7.16E-02	7.16E-02	1.43E-04	1.63E-05	5.86E-04
78	6.58E-04	4.70E-03	1.80E-04	8.86E-04	4.58E-02	4.58E-02	9.17E-05	1.04E-05	3.75E-04
79	1.09E-03	7.76E-03	2.97E-04	1.46E-03	7.55E-02	7.55E-02	1.51E-04	1.72E-05	6.18E-04
80	5.80E-04	4.14E-03	1.58E-04	7.80E-04	4.03E-02	4.03E-02	8.07E-05	9.19E-06	3.30E-04
81	7.93E-04	5.67E-03	2.17E-04	1.07E-03	5.52E-02	5.51E-02	1.11E-04	1.26E-05	4.51E-04
82	8.55E-04	6.11E-03	2.34E-04	1.15E-03	5.95E-02	5.94E-02	1.19E-04	1.36E-05	4.87E-04
83	8.38E-04	5.99E-03	2.29E-04	1.13E-03	5.83E-02	5.83E-02	1.17E-04	1.33E-05	4.77E-04
84	8.16E-04	5.83E-03	2.23E-04	1.10E-03	5.67E-02	5.67E-02	1.14E-04	1.29E-05	4.64E-04
85	8.83E-04	6.31E-03	2.41E-04	1.19E-03	6.14E-02	6.14E-02	1.23E-04	1.40E-05	5.03E-04
86	5.18E-04	3.70E-03	1.41E-04	6.96E-04	3.60E-02	3.60E-02	7.21E-05	8.20E-06	2.95E-04
87	1.15E-03	8.24E-03	3.15E-04	1.55E-03	8.02E-02	8.02E-02	1.61E-04	1.83E-05	6.56E-04

Supplementary Table S5d. HQ evaluation of Zn

Sample number	HQ_{ois} (Adult)	HQ_{ois} (Child)	HQ_{dcs} (Adult)	HQ_{dcs} (Child)
1	4.22E-03	3.02E-02	1.50E-05	7.38E-05
2	3.83E-03	2.73E-02	1.36E-05	6.69E-05
3	1.90E-02	1.35E-01	6.74E-05	3.32E-04
4	3.83E-03	2.73E-02	1.36E-05	6.69E-05
5	3.54E-03	2.53E-02	1.26E-05	6.20E-05

6	8.07E-03	5.77E-02	2.87E-05	1.41E-04
7	4.53E-03	3.24E-02	1.61E-05	7.92E-05
8	4.90E-03	3.50E-02	1.74E-05	8.56E-05
9	4.50E-03	3.22E-02	1.60E-05	7.87E-05
10	3.94E-03	2.81E-02	1.40E-05	6.89E-05
11	5.94E-03	4.24E-02	2.11E-05	1.04E-04
12	4.70E-03	3.36E-02	1.67E-05	8.22E-05
13	6.78E-03	4.84E-02	2.41E-05	1.19E-04
14	3.40E-03	2.43E-02	1.21E-05	5.95E-05
15	3.97E-03	2.83E-02	1.41E-05	6.94E-05
16	3.97E-03	2.83E-02	1.41E-05	6.94E-05
17	4.16E-03	2.97E-02	1.48E-05	7.28E-05
18	3.24E-03	2.31E-02	1.15E-05	5.66E-05
19	4.53E-03	3.24E-02	1.61E-05	7.92E-05
20	7.91E-03	5.65E-02	2.81E-05	1.38E-04
21	5.43E-03	3.88E-02	1.93E-05	9.50E-05
22	4.39E-03	3.14E-02	1.56E-05	7.68E-05
23	5.88E-03	4.20E-02	2.09E-05	1.03E-04
24	8.61E-03	6.15E-02	3.06E-05	1.51E-04
25	3.60E-03	2.57E-02	1.28E-05	6.30E-05
26	6.98E-03	4.98E-02	2.48E-05	1.22E-04
27	3.71E-03	2.65E-02	1.32E-05	6.49E-05
28	1.13E-02	8.06E-02	4.01E-05	1.97E-04
29	3.38E-03	2.41E-02	1.20E-05	5.90E-05
30	8.44E-03	6.03E-02	3.00E-05	1.48E-04
31	7.68E-03	5.49E-02	2.73E-05	1.34E-04
32	1.04E-02	7.42E-02	3.69E-05	1.82E-04
33	9.57E-03	6.83E-02	3.40E-05	1.67E-04
34	7.03E-03	5.03E-02	2.50E-05	1.23E-04
35	4.47E-03	3.20E-02	1.59E-05	7.82E-05
36	5.57E-03	3.98E-02	1.98E-05	9.74E-05
37	6.02E-03	4.30E-02	2.14E-05	1.05E-04
38	4.16E-03	2.97E-02	1.48E-05	7.28E-05
39	4.05E-03	2.89E-02	1.44E-05	7.08E-05
40	3.77E-03	2.69E-02	1.34E-05	6.59E-05
41	9.37E-03	6.69E-02	3.33E-05	1.64E-04
42	5.01E-03	3.58E-02	1.78E-05	8.76E-05
43	1.41E-02	1.01E-01	5.02E-05	2.47E-04
44	7.85E-03	5.61E-02	2.79E-05	1.37E-04
45	8.58E-03	6.13E-02	3.05E-05	1.50E-04
46	4.39E-03	3.14E-02	1.56E-05	7.68E-05
47	4.42E-03	3.16E-02	1.57E-05	7.72E-05
48	3.88E-03	2.77E-02	1.38E-05	6.79E-05
49	5.88E-03	4.20E-02	2.09E-05	1.03E-04

50	4.90E-03	3.50E-02	1.74E-05	8.56E-05
51	3.69E-03	2.63E-02	1.31E-05	6.45E-05
52	8.44E-03	6.03E-02	3.00E-05	1.48E-04
53	4.75E-03	3.40E-02	1.69E-05	8.31E-05
54	4.50E-03	3.22E-02	1.60E-05	7.87E-05
55	3.52E-03	2.51E-02	1.25E-05	6.15E-05
56	6.10E-03	4.36E-02	2.17E-05	1.07E-04
57	3.32E-03	2.37E-02	1.18E-05	5.81E-05
58	3.15E-03	2.25E-02	1.12E-05	5.51E-05
59	4.30E-03	3.08E-02	1.53E-05	7.53E-05
60	1.65E-02	1.18E-01	5.87E-05	2.89E-04
61	6.36E-03	4.54E-02	2.26E-05	1.11E-04
62	7.15E-03	5.11E-02	2.54E-05	1.25E-04
63	2.81E-03	2.01E-02	1.00E-05	4.92E-05
64	6.72E-03	4.80E-02	2.39E-05	1.18E-04
65	3.74E-03	2.67E-02	1.33E-05	6.54E-05
66	5.74E-03	4.10E-02	2.04E-05	1.00E-04
67	1.52E-02	1.09E-01	5.42E-05	2.67E-04
68	4.42E-03	3.16E-02	1.57E-05	7.72E-05
69	5.82E-03	4.16E-02	2.07E-05	1.02E-04
70	4.98E-03	3.56E-02	1.77E-05	8.71E-05
71	7.51E-03	5.37E-02	2.67E-05	1.31E-04
72	4.84E-03	3.46E-02	1.72E-05	8.46E-05
73	4.78E-03	3.42E-02	1.70E-05	8.36E-05
74	4.73E-03	3.38E-02	1.68E-05	8.27E-05
75	9.96E-03	7.12E-02	3.54E-05	1.74E-04
76	7.17E-03	5.13E-02	2.55E-05	1.25E-04
77	3.85E-03	2.75E-02	1.37E-05	6.74E-05
78	3.46E-03	2.47E-02	1.23E-05	6.05E-05
79	5.54E-03	3.96E-02	1.97E-05	9.69E-05
80	5.18E-03	3.70E-02	1.84E-05	9.05E-05
81	4.64E-03	3.32E-02	1.65E-05	8.12E-05
82	4.14E-03	2.95E-02	1.47E-05	7.23E-05
83	3.40E-03	2.43E-02	1.21E-05	5.95E-05
84	3.40E-03	2.43E-02	1.21E-05	5.95E-05
85	4.22E-03	3.02E-02	1.50E-05	7.38E-05
86	4.78E-03	3.42E-02	1.70E-05	8.36E-05
87	5.94E-03	4.24E-02	2.11E-05	1.04E-04

Supplementary Table S5e. HQ and CR evaluation of Ni

Sample number	HQ_{ois} (Adult)	HQ_{ois} (Child)	HQ_{dcs} (Adult)	HQ_{dcs} (Child)	HQ_{pis} (Adult)	HQ_{pis} (Child)	CR_{pis}
1	3.54E-04	2.53E-03	2.36E-03	1.16E-02	2.74E-02	2.74E-02	6.24E-07
2	3.54E-04	2.53E-03	2.36E-03	1.16E-02	2.74E-02	2.74E-02	6.24E-07

3	4.90E-04	3.50E-03	3.26E-03	1.61E-02	3.78E-02	3.78E-02	8.62E-07
4	4.95E-04	3.54E-03	3.30E-03	1.62E-02	3.82E-02	3.82E-02	8.72E-07
5	4.11E-04	2.93E-03	2.74E-03	1.35E-02	3.17E-02	3.17E-02	7.23E-07
6	4.33E-04	3.10E-03	2.89E-03	1.42E-02	3.34E-02	3.35E-02	7.63E-07
7	4.39E-04	3.14E-03	2.92E-03	1.44E-02	3.39E-02	3.39E-02	7.73E-07
8	3.71E-04	2.65E-03	2.47E-03	1.22E-02	2.87E-02	2.87E-02	6.54E-07
9	4.78E-04	3.42E-03	3.19E-03	1.57E-02	3.69E-02	3.69E-02	8.42E-07
10	5.23E-04	3.74E-03	3.49E-03	1.72E-02	4.04E-02	4.04E-02	9.21E-07
11	4.78E-04	3.42E-03	3.19E-03	1.57E-02	3.69E-02	3.69E-02	8.42E-07
12	4.67E-04	3.34E-03	3.11E-03	1.53E-02	3.60E-02	3.61E-02	8.22E-07
13	6.25E-04	4.46E-03	4.16E-03	2.05E-02	4.82E-02	4.82E-02	1.10E-06
14	5.01E-04	3.58E-03	3.34E-03	1.64E-02	3.87E-02	3.87E-02	8.81E-07
15	5.74E-04	4.10E-03	3.82E-03	1.88E-02	4.43E-02	4.43E-02	1.01E-06
16	4.16E-04	2.97E-03	2.77E-03	1.37E-02	3.21E-02	3.22E-02	7.33E-07
17	5.01E-04	3.58E-03	3.34E-03	1.64E-02	3.87E-02	3.87E-02	8.81E-07
18	4.73E-04	3.38E-03	3.15E-03	1.55E-02	3.65E-02	3.65E-02	8.32E-07
19	5.12E-04	3.66E-03	3.41E-03	1.68E-02	3.95E-02	3.95E-02	9.01E-07
20	4.90E-04	3.50E-03	3.26E-03	1.61E-02	3.78E-02	3.78E-02	8.62E-07
21	4.73E-04	3.38E-03	3.15E-03	1.55E-02	3.65E-02	3.65E-02	8.32E-07
22	5.51E-04	3.94E-03	3.67E-03	1.81E-02	4.26E-02	4.26E-02	9.71E-07
23	4.90E-04	3.50E-03	3.26E-03	1.61E-02	3.78E-02	3.78E-02	8.62E-07
24	5.57E-04	3.98E-03	3.71E-03	1.83E-02	4.30E-02	4.30E-02	9.81E-07
25	4.50E-04	3.22E-03	3.00E-03	1.48E-02	3.47E-02	3.48E-02	7.92E-07
26	8.21E-04	5.87E-03	5.47E-03	2.69E-02	6.34E-02	6.34E-02	1.45E-06
27	4.45E-04	3.18E-03	2.96E-03	1.46E-02	3.43E-02	3.43E-02	7.82E-07
28	1.70E-03	1.22E-02	1.14E-02	5.59E-02	1.32E-01	1.32E-01	3.00E-06
29	4.95E-04	3.54E-03	3.30E-03	1.62E-02	3.82E-02	3.82E-02	8.72E-07
30	4.78E-04	3.42E-03	3.19E-03	1.57E-02	3.69E-02	3.69E-02	8.42E-07
31	4.78E-04	3.42E-03	3.19E-03	1.57E-02	3.69E-02	3.69E-02	8.42E-07
32	5.57E-04	3.98E-03	3.71E-03	1.83E-02	4.30E-02	4.30E-02	9.81E-07
33	6.25E-04	4.46E-03	4.16E-03	2.05E-02	4.82E-02	4.82E-02	1.10E-06
34	5.85E-04	4.18E-03	3.90E-03	1.92E-02	4.52E-02	4.52E-02	1.03E-06
35	4.95E-04	3.54E-03	3.30E-03	1.62E-02	3.82E-02	3.82E-02	8.72E-07
36	5.01E-04	3.58E-03	3.34E-03	1.64E-02	3.87E-02	3.87E-02	8.81E-07
37	4.73E-04	3.38E-03	3.15E-03	1.55E-02	3.65E-02	3.65E-02	8.32E-07
38	4.11E-04	2.93E-03	2.74E-03	1.35E-02	3.17E-02	3.17E-02	7.23E-07
39	4.84E-04	3.46E-03	3.22E-03	1.59E-02	3.73E-02	3.74E-02	8.52E-07
40	4.78E-04	3.42E-03	3.19E-03	1.57E-02	3.69E-02	3.69E-02	8.42E-07
41	4.84E-04	3.46E-03	3.22E-03	1.59E-02	3.73E-02	3.74E-02	8.52E-07
42	5.06E-04	3.62E-03	3.37E-03	1.66E-02	3.91E-02	3.91E-02	8.91E-07
43	6.92E-04	4.94E-03	4.61E-03	2.27E-02	5.34E-02	5.34E-02	1.22E-06
44	4.50E-04	3.22E-03	3.00E-03	1.48E-02	3.47E-02	3.48E-02	7.92E-07
45	5.35E-04	3.82E-03	3.56E-03	1.75E-02	4.13E-02	4.13E-02	9.41E-07
46	4.84E-04	3.46E-03	3.22E-03	1.59E-02	3.73E-02	3.74E-02	8.52E-07
47	4.39E-04	3.14E-03	2.92E-03	1.44E-02	3.39E-02	3.39E-02	7.73E-07
48	3.09E-04	2.21E-03	2.06E-03	1.01E-02	2.39E-02	2.39E-02	5.45E-07
49	3.99E-04	2.85E-03	2.66E-03	1.31E-02	3.08E-02	3.09E-02	7.03E-07

50	2.93E-04	2.09E-03	1.95E-03	9.59E-03	2.26E-02	2.26E-02	5.15E-07
51	4.61E-04	3.30E-03	3.07E-03	1.51E-02	3.56E-02	3.56E-02	8.12E-07
52	4.16E-04	2.97E-03	2.77E-03	1.37E-02	3.21E-02	3.22E-02	7.33E-07
53	3.49E-04	2.49E-03	2.32E-03	1.14E-02	2.69E-02	2.69E-02	6.14E-07
54	3.88E-04	2.77E-03	2.59E-03	1.27E-02	3.00E-02	3.00E-02	6.83E-07
55	3.88E-04	2.77E-03	2.59E-03	1.27E-02	3.00E-02	3.00E-02	6.83E-07
56	2.64E-04	1.89E-03	1.76E-03	8.67E-03	2.04E-02	2.04E-02	4.65E-07
57	3.99E-04	2.85E-03	2.66E-03	1.31E-02	3.08E-02	3.09E-02	7.03E-07
58	4.05E-04	2.89E-03	2.70E-03	1.33E-02	3.13E-02	3.13E-02	7.13E-07
59	4.50E-04	3.22E-03	3.00E-03	1.48E-02	3.47E-02	3.48E-02	7.92E-07
60	5.57E-04	3.98E-03	3.71E-03	1.83E-02	4.30E-02	4.30E-02	9.81E-07
61	4.39E-04	3.14E-03	2.92E-03	1.44E-02	3.39E-02	3.39E-02	7.73E-07
62	4.56E-04	3.26E-03	3.04E-03	1.49E-02	3.52E-02	3.52E-02	8.02E-07
63	3.54E-04	2.53E-03	2.36E-03	1.16E-02	2.74E-02	2.74E-02	6.24E-07
64	4.16E-04	2.97E-03	2.77E-03	1.37E-02	3.21E-02	3.22E-02	7.33E-07
65	3.49E-04	2.49E-03	2.32E-03	1.14E-02	2.69E-02	2.69E-02	6.14E-07
66	3.83E-04	2.73E-03	2.55E-03	1.25E-02	2.95E-02	2.95E-02	6.73E-07
67	5.35E-04	3.82E-03	3.56E-03	1.75E-02	4.13E-02	4.13E-02	9.41E-07
68	4.45E-04	3.18E-03	2.96E-03	1.46E-02	3.43E-02	3.43E-02	7.82E-07
69	4.50E-04	3.22E-03	3.00E-03	1.48E-02	3.47E-02	3.48E-02	7.92E-07
70	4.05E-04	2.89E-03	2.70E-03	1.33E-02	3.13E-02	3.13E-02	7.13E-07
71	4.84E-04	3.46E-03	3.22E-03	1.59E-02	3.73E-02	3.74E-02	8.52E-07
72	4.67E-04	3.34E-03	3.11E-03	1.53E-02	3.60E-02	3.61E-02	8.22E-07
73	3.77E-04	2.69E-03	2.51E-03	1.24E-02	2.91E-02	2.91E-02	6.64E-07
74	3.43E-04	2.45E-03	2.29E-03	1.13E-02	2.65E-02	2.65E-02	6.04E-07
75	5.12E-04	3.66E-03	3.41E-03	1.68E-02	3.95E-02	3.95E-02	9.01E-07
76	5.68E-04	4.06E-03	3.79E-03	1.86E-02	4.39E-02	4.39E-02	1.00E-06
77	5.23E-04	3.74E-03	3.49E-03	1.72E-02	4.04E-02	4.04E-02	9.21E-07
78	3.49E-04	2.49E-03	2.32E-03	1.14E-02	2.69E-02	2.69E-02	6.14E-07
79	4.67E-04	3.34E-03	3.11E-03	1.53E-02	3.60E-02	3.61E-02	8.22E-07
80	4.28E-04	3.06E-03	2.85E-03	1.40E-02	3.30E-02	3.30E-02	7.53E-07
81	1.91E-04	1.37E-03	1.27E-03	6.27E-03	1.48E-02	1.48E-02	3.37E-07
82	2.14E-04	1.53E-03	1.42E-03	7.01E-03	1.65E-02	1.65E-02	3.76E-07
83	2.25E-04	1.61E-03	1.50E-03	7.38E-03	1.74E-02	1.74E-02	3.96E-07
84	1.86E-04	1.33E-03	1.24E-03	6.09E-03	1.43E-02	1.43E-02	3.27E-07
85	2.08E-04	1.49E-03	1.39E-03	6.83E-03	1.61E-02	1.61E-02	3.66E-07
86	1.86E-04	1.33E-03	1.24E-03	6.09E-03	1.43E-02	1.43E-02	3.27E-07
87	3.88E-04	2.77E-03	2.59E-03	1.27E-02	3.00E-02	3.00E-02	6.83E-07

Supplementary Table S5f. HQ evaluation of Cu

Sample number	HQ_{ois} (Adult)	HQ_{ois} (Child)
1	8.86E-03	6.33E-02
2	1.01E-02	7.24E-02
3	1.56E-02	1.12E-01
4	1.20E-02	8.59E-02
5	1.35E-02	9.65E-02

6	1.35E-02	9.65E-02
7	9.92E-03	7.09E-02
8	1.01E-02	7.24E-02
9	8.65E-03	6.18E-02
10	8.44E-03	6.03E-02
11	6.33E-03	4.52E-02
12	7.60E-03	5.43E-02
13	1.54E-02	1.10E-01
14	7.39E-03	5.28E-02
15	1.03E-02	7.39E-02
16	6.96E-03	4.97E-02
17	7.81E-03	5.58E-02
18	6.96E-03	4.97E-02
19	8.44E-03	6.03E-02
20	1.31E-02	9.35E-02
21	8.23E-03	5.88E-02
22	7.81E-03	5.58E-02
23	7.81E-03	5.58E-02
24	1.58E-02	1.13E-01
25	5.70E-03	4.07E-02
26	1.54E-02	1.10E-01
27	6.12E-03	4.37E-02
28	6.94E-02	4.96E-01
29	6.33E-03	4.52E-02
30	1.22E-02	8.74E-02
31	3.99E-02	2.85E-01
32	1.69E-01	1.21E+00
33	2.17E-02	1.55E-01
34	1.43E-02	1.03E-01
35	9.50E-03	6.78E-02
36	1.90E-02	1.36E-01
37	8.44E-03	6.03E-02
38	6.12E-03	4.37E-02
39	6.75E-03	4.82E-02
40	8.23E-03	5.88E-02
41	1.10E-02	7.84E-02
42	9.50E-03	6.78E-02
43	5.19E-02	3.71E-01
44	3.42E-02	2.44E-01
45	5.91E-02	4.22E-01
46	1.10E-02	7.84E-02
47	7.81E-03	5.58E-02
48	9.07E-03	6.48E-02
49	1.16E-02	8.29E-02
50	8.23E-03	5.88E-02
51	9.07E-03	6.48E-02

52	1.22E-02	8.74E-02
53	8.44E-03	6.03E-02
54	8.23E-03	5.88E-02
55	1.35E-02	9.65E-02
56	1.62E-02	1.16E-01
57	8.02E-03	5.73E-02
58	6.33E-03	4.52E-02
59	7.39E-03	5.28E-02
60	1.03E-02	7.39E-02
61	7.17E-03	5.13E-02
62	1.33E-02	9.50E-02
63	5.49E-03	3.92E-02
64	9.07E-03	6.48E-02
65	6.54E-03	4.67E-02
66	6.96E-03	4.97E-02
67	1.28E-01	9.17E-01
68	1.08E-02	7.69E-02
69	1.06E-02	7.54E-02
70	7.39E-03	5.28E-02
71	1.20E-02	8.59E-02
72	1.22E-02	8.74E-02
73	1.01E-02	7.24E-02
74	9.50E-03	6.78E-02
75	4.68E-02	3.35E-01
76	1.52E-02	1.09E-01
77	1.39E-02	9.95E-02
78	6.96E-03	4.97E-02
79	1.60E-02	1.15E-01
80	1.60E-02	1.15E-01
81	7.17E-03	5.13E-02
82	8.02E-03	5.73E-02
83	8.44E-03	6.03E-02
84	6.96E-03	4.97E-02
85	7.81E-03	5.58E-02
86	6.96E-03	4.97E-02
87	1.46E-02	1.04E-01

Supplementary Table S6. Landscape plants with the remediation ability of heavy metal contamination.

Plant classification	Plant	Metal remediation	Reference
Arbor	Cinnamomum austrosinense H. T. Chang	Pb	1
	Broussonetia papyrifera	Zn、Cu	2
	Liquidambar formosana	Pb	3
	Rhus chinensis Mil	Pb	4
	Willow	Cu, Pb, Zn	5
	Koelreuteria paniculata	Pb、Zn	6

	Paulownia fortunei	Pb、Zn	7
	Betula platyphylla	Zn	8
	Ilex chinensis	Pb、Zn	9
Shrub	Rosa rugosa	Cr、Ni、Cu、Pb、	10
	Lagerstroemia indica	Ni	11
	Pelargonium hortorum	Cr、Ni	10
	Rubus setchuenensis Bureau et Franch.	Pb	12
	Nerium oleander	Pb	13
	Ligustrum lucidum	Cr	14
	Amorpha fruticosa	Zn，Cu 、Pb	15
	Ligustrum vicaryi	Cu、Zn、Pb	16
	Platycladus orientalis	Cr、Pb	17
Ground cover	Tagetes erecta	Cr、Zn、Cu	18
	Vetiveria zizanioides	Cr、Pb、Ni、Zn	19
	Polygonum capitatum	Cr、Ni、Cu	12
	Rhododendron simsii	Pb、Zn、Cr	20
	Chrysanthemum indicum	Cr、Ni、Cu	12
	Miscanthus	Cr、Ni、Cu	12
	Conyza canadensis	Cr、Ni、Cu	12
	Arabidopsis thaliana	Pb	21
	Miscanthus sinensis	As	19
	Phragmites communis	Pb	19
	Helianthus annuus	Pb	22
	Calendula officinalis	Pb、Zn、Ni、Cr	21
	Trollius chinensis	Pb	23
	Sedum alfredii	Zn	23
	Senecio scandens	Zn	12
	Pteris vittata	As	24
	Iris lacteaPall. var.chinensis	Pb	25
	Nephrolepis auriculata	As.Pb	26
	Chlorophytum comosum f. variegata	Cd	27
	Commelina communis	Cu	28
	Pennisetum alopecuroides	Cr	29
	Arundo donax var. versicolor Stokes	Pb、Zn、Cr	30
	Artemisia vulgaris	Ni	31
	Pteris vittata	As、Pb	32
Turfgrasses	Ophiopogon japonicus	Cu, Zn	33
	Festuca elata Keng ex E. Alexeev	Cu, Pb, Zn	5
	Lolium perenne	Pb	34
	Cynodon dactylon	Pb, Zn	35

References

1. Chang, X. , et al. Occurrence, source apportionment, plant bioaccumulation and human exposure of legacy and emerging per- and polyfluoroalkyl substances in soil and plant leaves near a landfill in China. *Science of The Total Environment.* **776,** 145731 (2021).
2. Zhao, X. , et al. The evaluation of heavy metal accumulation and application of a comprehensive bio-concentration index for woody species on contaminated sites in Hunan, China. *Environmental Science and Pollution Research.* **21(7)**, 5076-5085 (2014).
3. Shi, X. , Chen, YTi. , Wang, SF. , Pan, HW. , & Sun, HJ. Phytoremediation potential of transplanted

bare-root seedlings of trees for lead/zinc and copper mine tailings. *International Journal of Phytoremediation.* **18(11)**, 1155-1163 (2016).

4. Zhou, C. , et al. Bioaccumulation and detoxification mechanisms for lead uptake identified in Rhus chinensis Mill. seedlings. *Ecotoxicology & Environmental Safety.* **142(aug.)**, 59 (2017).

5. Roy, S. , et al. Phytoremediation of heavy metal and PAH-contaminated brownfield sites. *Plant and Soil.* **272(1-2)**, 277-290 (2005).

6. Wang, J. , Chen, X. , Yan, W. , Ning, C. , & Gsell, T. Both artificial root exudates and natural Koelreuteria paniculata exudates modify bacterial community structure and enhance phenanthrene biodegradation in contaminated soils. *Chemosphere.* **263,** 128041 (2020).

7. Han, L. , Chen, Y. , Chen, M. , Wu, Y. , & Liu, Z. Mushroom residue modification enhances phytoremediation potential of Paulownia fortunei to lead-zinc slag. *Chemosphere.* **253(1)**, 126774 (2020).

8. Gallagher, FJ. , Pechmann, I. , Bogden, JD. , Grabosky, J. , & Weis, P. Soil metal concentrations and vegetative assemblage structure in an urban brownfield. *Environmental Pollution.* **153(2)**, 351-361 (2008).

9. Ward, NI. , Brooks, RR. , Roberts, E. , & Boswell, CR. Heavy-metal pollution from automotive emissions and its effect on roadside soils and pasture species in New Zealand. *Environmental Science & Technology.* **11(9)**, 917-920 (1977).

10. Chand, S. , Singh, G. , Kumari, R. , & Patra, DD. Performance of rose scented geranium (Pelargonium graveolens) in heavy metal polluted soil vis-à-vis phytoaccumulation of metals. *Int J Phytoremediation.* **18(8)**, 754-760 (2016).

11. Periasamy, K. , & Namasivayam, C. Removal of nickel(II) from aqueous solution and nickel plating industry wastewater using an agricultural waste: Peanut hulls. *Waste Management.* **15(1)**, 63-68 (1995).

12. Wu, B. , Peng, H. , Sheng, M. , Luo, H. , & Xu, H. Evaluation of phytoremediation potential of native dominant plants and spatial distribution of heavy metals in abandoned mining area in Southwest China. *Ecotoxicology and Environmental Safety.* **220,** 112368 (2021).

13. Ibrahim, N. , & Afandi, GE. Phytoremediation uptake model of heavy metals (Pb, Cd and Zn) in soil using Nerium oleander. *Heliyon.* **6(7)**, 1-7 (2020).

14. Zhang. C., et al. Bioaccumulation of zinc, lead, copper, and cadmium from contaminated sediments by native plant species and Acrida cinerea in South China. *Environmental Monitoring and Assessment.* **186(3)**, 1735-1745 (2014).

15. Zaccheo, P. , & Pasta, C. Ammonium nutrition as a strategy for cadmium mobilisation in the rhizosphere of sunflower. *Plant&Soil.* **283**, 43-56 (2007).

16. Hu, Y. , Wang, D. , Wei, L. , Zhang, X. , & Song, B. Bioaccumulation of heavy metals in plant leaves from Yanan city of the Loess Plateau, China. *Ecotoxicology & Environmental Safety.* **110(dec.)**, 82-88 (2014).

17. Mohammadhosseini, M. , Akbarzadeh, A. , Hashemi-Moghaddam, H. , Bahmanpour, H. , & Lotfi, S. The Relationship Between Chemical Composition of the Essential Oils of Platycladus orientalis (L.) Franco and Soils Contamination in National Oil Company of Shahrood, Iran. *Journal of essential oil-bearing plants JEOP.* **20(5)**, 1209-1225 (2017).

18. Choudhury, MR. , Islam, MS. , Ahmed, ZU. , & Nayar, F. Phytoremediation of heavy metal contaminated buriganga riverbed sediment by Indian mustard and marigold plants. *Environmental Progress & Sustainable Energy.* **35(1)**, 117-124 (2016).

19. Shah, V. , & Daverey, A. Phytoremediation: A multidisciplinary approach to clean up heavy metal contaminated soil. *Environmental Technology & Innovation.* **18**, 100774 (2020).
20. Zu, Y. , et al. Hyperaccumulation of Pb, Zn and Cd in herbaceous grown on lead–zinc mining area in Yunnan, China. *Environment international.* **31(5)**, 755-762 (2005).
21. Saraswat, S. , & Rai, JPN. Phytoextraction potential of six plant species grown in multimetal contaminated soil. *Chemistry and Ecology.* **25(1)**, 1-11 (2009).
22. Alaboudi, KA. , Ahmed, B. , & Brodie, G. Phytoremediation of Pb and Cd contaminated soils by using sunflower (Helianthus annuus) plant. *Annals of Agricultural Sciences.* **63(1)**, 123-127 (2018).
23. Xu, ZH. , Jiang, X. , & Zhu, YW. *Plant Resources for Phytoremediation of Heavy Metal contaminated Soils*. Science (2018).
24. Lena, Q, Ma. , Kenneth, M, Komar. , Cong, Tu. , & Weihua, Zhang. A fern that hyperaccumulates arsenic. *Nature.* **409(6820)**, 579 (2001).
25. Han, YL. , Huang, SZ. , Gu, JG. , Qiu, S. , & Chen, JM. Tolerance and accumulation of lead by species of Iris L. *Ecotoxicology.* **17(8)**, 853-859 (2008).
26. Deng, T. , Liao, M. , Pan, Z. , & Liu, C. Phytoremediation of Nephrolepis auriculata (L.) for arsenic, mercury, lead and cadmium in the multiple contaminated soils. *Chinese Journal of Geochemistry.* **25(s1)**, 95-95 (2006).
27. Wang, Y. , Yan, A. , Dai, J. , & Wu, WD. Accumulation and tolerance characteristics of cadmium in Chlorophytum comosum: a popular ornamental plant and potential Cd hyperaccumulator. *Environmental Monitoring&Assessment.* **184,** 929-937 (2012).
28. Dwivedi, S. , et al. Bioremediation potential of genus Portulaca L. collected from industrial areas in Vadodara, Gujarat, India. *Clean Technologies & Environmental Policy.* **14(2)**, 223-228 (2012).
29. Lassalle, G. , Gassend, V. , Michaudel, G. , Hédacq, R. , & Credoz, A. A multicriteria approach for assessing the recovery of soil functions following high-temperature remediation of hydrocarbons. *Science of The Total Environment.* **775(25)**,145891 (2021).
30. Sun, H. , Wang, Z. , Gao, P. , & Liu, P. Selection of aquatic plants for phytoremediation of heavy metal in electroplate wastewater. *Acta Physiologiae Plantarum.* **35,** 355-364 (2013).
31. Va, A. , et al. Phytoremediation potential of twelve wild plant species for toxic elements in a contaminated soil. *Environment International.* **146**, 106233 (2021).
32. Salido, AL. , Hasty, KL. , Lim, JM. , & Butcher, DJ. Phytoremediation of arsenic and lead in contaminated soil using Chinese brake ferns (Pteris vittata) and Indian mustard (Brassica juncea). *Int J Phytoremediation.* **5(2)**, 89-103 (2003).
33. Ma, W. , Zhao, B. , & Ma, J. Comparison of heavy metal accumulation ability in rainwater by 10 sponge city plant species. *Environmental Science and Pollution Research.* **26(26)**, 26733-26747 (2019).
34. Xu, P. , et al. The effect of biochar and crop straws on heavy metal bioavailability and plant accumulation in a Cd and Pb polluted soil. *Ecotoxicology & Environmental Safety.* **132(Oct.)**, 94-100 (2016).
35. Zhan, FD. , et al. Effects of arbuscular mycorrhizal fungi on the growth and heavy metal accumulation of bermudagrass [Cynodon dactylon (L.) Pers.] grown in a lead-zinc mine wasteland. *International journal of phytoremediation.* **21(9)**, 849-856 (2019).
